# Stem borers revisited: Host resistance, tolerance, and vulnerability determine levels of field damage from a complex of Asian rice stemborers

**DOI:** 10.1016/j.cropro.2020.105513

**Published:** 2021-04

**Authors:** Finbarr G. Horgan, Angelita M. Romena, Carmencita C. Bernal, Maria Liberty P. Almazan, Angelee Fame Ramal

**Affiliations:** a EcoLaVerna Integral Restoration Ecology, Bridestown, Kildinan, Co. Cork, Ireland; b Universidad Católica del Maule, Facultad de Ciencias Agrarias y Forestales, Escuela de Agronomía, Casilla 7-D, Curicó, Chile; c Environment and Sustainable Resource Management, University College Dublin, Belfield, Dublin 4, Ireland; d International Rice Research Institute, DAPO Box 7777, Metro Manila, Philippines; e School of Environmental Science and Management, University of the Philippines, Los Baños, 4030, Laguna, Philippines

**Keywords:** *Chilo*, Nitrogen, Preference-performance, Resistance, Rice breeding, *Scirpophaga*, Tolerance, Vulnerability

## Abstract

Yield losses from rice stem borers depend on the nature of the rice variety, the timing of attack, and the composition of the stem borer species assemblage. This study uses a range of phenotyping methods to distinguish different categories of herbivore-rice interaction that determine relative damage levels (dead heart and whitehead-panicles) and yield losses to varieties exposed to stem borers. Phenotyping studies were conducted in a greenhouse, screen house and field using two stem borer species (*Scirpophaga incertulas* [yellow stem borer - YSB] and *Chilo suppressalis* [striped stem borer - SSB]) and 12 rice lines. Only YSB displayed oviposition preferences across rice varieties. Both stem borer species performed better (greater survival, shorter development times, heavier pupae) on rice at vegetative compared to reproductive growth stages, and SSB was less capable than YSB of developing on rice at reproductive growth stages. Stem borer larval survival, body weight, development time, and tiller damage across different rice varieties in greenhouse experiments was poorly correlated between the two stem borer species and for each of the species on rice at two different plant growth stages (vegetative and reproductive). In contrast, rice tillering and yield responses to the two stem borer species were often highly correlated, but only when plants were attacked at the reproductive stage. Short-term, controlled experiments revealed aspects of host resistance and relative changes in plant biomass and yield in response to damage (condition change). However, the results from controlled environments and field-plots were not generally correlated because plant vulnerability, i.e., relative exposure to stem borer attack due to crop duration, largely determined field damage. We recommend that phenotyping studies should differentiate between the resistance, tolerance and vulnerability of rice lines to complexes of stem borers in a given region. Single stem borer species experiments under controlled environments are useful to gain knowledge of the nature of rice-stem borer interactions; however, field testing with comparative treatments, particularly under high soil fertilizer levels that increase plant attractiveness, are better for assessing the relative propensities of rice varieties to incur damage and yield losses due to complexes of stem borers.

## Highlights

• Stem borer-rice interactions are categorized as relative resistance, and tolerance.
• Phenotyping with 2 stem borers on 12 rice lines was used to predict field damage.
• Results from tests under controlled conditions poorly correlated with field results.
• Vulnerability, determined by crop duration, had a large impact on field damage.
• Field tests that distinguish resistance, tolerance and vulnerability are recommended.

## Graphical abstract

**Figure:**
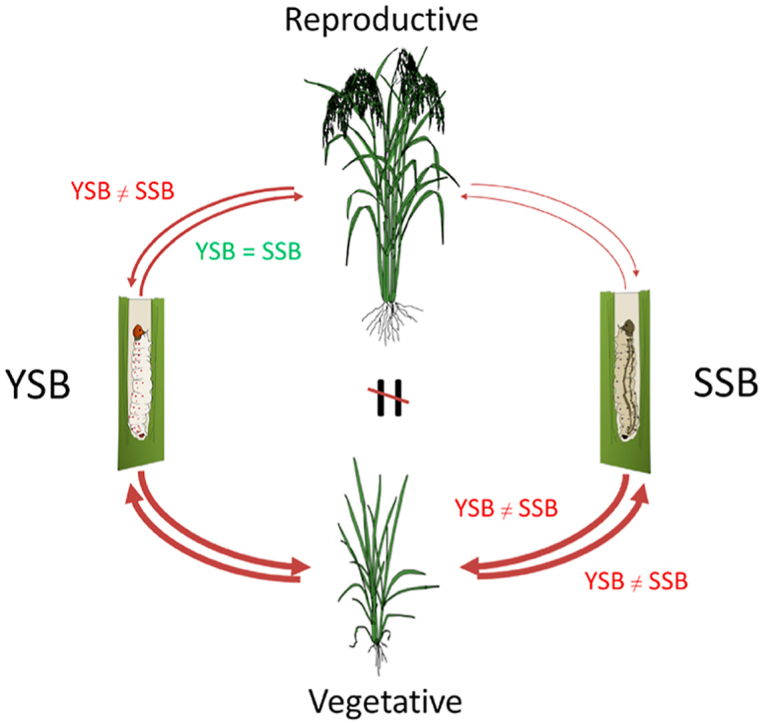

## 1 Introduction

Stem borers cause damage to cereal crops worldwide (Lawani, 1982; Heinrichs, 1985; Kfir et al., 2002). A range of species that includes moths (Lepidoptera) and flies (Diptera) can cause yield losses of >70% to rice in some regions (e.g., *Scirpophaga innotata* in Java: Rubia Sanchez et al., 1997; *Diatraea saccharalis* in Brazil and southern USA: Ferreira et al., 1998; Hamm et al., 2012; the stem borer complex in Nigeria: Ogah, 2013) and are one of the principal targets of insecticide applications (Rubia Sanchez et al., 1997; Bandong and Litsinger, 2005). This is partly because the damage stem borers cause has a high visual impact (they cause rice panicles to turn white due to empty grains – known as ‘whitehead’ panicles) and because their damage is often most visible at about the time of rice harvest (Rubia et al., 1989). In many cases, particularly when stem borers attack young, tillering rice plants, yield losses are minimal because the plants can compensate for damage by producing new tillers or overcompensate by redirecting resources to produce larger and more rice grain (Rubia et al., 1996; Horgan et al., 2016). Often, these compensatory mechanisms do not reduce the signs of damage and are therefore unperceived by farmers. Furthermore, the death or reductions in biomass of rice tillers due to stem borer feeding, even in cases of compensation, will sometimes cause a delay in grain maturation that ultimately leads to losses during harvest (e.g., *Diopsis longicornis* and *Maliarpha* spp.: Alghali, 1983; Bianchi et al., 1993). Rice yield losses due to stem borers and the choice by farmers to predominantly use insecticides to control stem borers, demonstrate a continuing need to develop sustainable stem borer management strategies.

In recent years, there has been a noted decline in research on rice-stem borer interactions, including on conventional host-plant resistance for sustainable herbivore management (Horgan, 2017). This has been partly due to the polyphagous nature of many stem borers and the difficulties in identifying stem borer-specific resistance genes in rice (Lu et al., 2017, 2018). Research interest in host resistance to stem borers has also waned in the face of the tremendous attention paid to developing transgenic rice with Bt or other anti-herbivore toxins (Horgan, 2017). However, there have been several complications with the development, deployment and acceptance of transgenic crops (Carrière et al., 2019; Sosa et al., 2019). Therefore, the use of native sources of stem borer resistance deserves renewed attention, particularly in Asia. Already, national and international agricultural research and advisory centers will often indicate categories of stem borer-rice interaction (e.g., susceptible [S], moderately susceptible [MS], or resistant [R]) for registered rice varieties, but without supporting information on screening methods or best crop management practices (Khush and Virk, 2005). A number of authors and research centers have presented protocols to screen rice for resistance to stem borers (Heinrichs, 1985; Togola et al., 2011; Elanchezhyan and Arumugachamy, 2015). However, there is often little information to support the choice of different phenotyping methods, and a number of phenotyping methods seem more suited to the discovery of gene-for-gene resistance sources, such as those involved in resistance to sap sucking-insects, rather than the polygenic resistance and tolerance mechanisms that influence stem borer-rice interactions.

The relative damage from insect herbivores to varieties of crop plants is influenced by a range of factors that include host plant resistance, plant tolerance to attack, and vulnerability (Table 1). Each of these factors are relative conditions. For example, host resistance is the relative capacity of a plant to deter an herbivore attack (antixenosis) or reduce the fitness of an herbivore (antibiosis) through physical or biochemical means, compared to a susceptible variety (Heinrichs, 1985, Horgan, 2017, Kalode et al., 1987). Tolerance is the relative capacity of a plant to withstand an herbivore attack and to continue to grow and develop in the face of damage or compensate for the damage, thereby gaining a high biomass or greater reproductive output (e.g., cereal yield), relative to a non-tolerant plant under equal herbivore pressures (Horgan, 2017, Horgan and Crisol, 2013) (Table 1). Tolerance will often depend on a plant's size and growth rates, and is influenced by resource availability. For example, plants grown under high nitrogen levels are often more tolerant of herbivore damage than plants grown under low nitrogen (Horgan et al., 2016, Horgan et al., 2018, Horgan et al., 2019). Plant vulnerability is seldom considered in studies of arthropod-plant interactions; however, vulnerability may be a significant determinant of plant damage and is likely to gain increasing importance under the influence of global climate change where populations of insect herbivores have increased voltinism under warmer temperatures (Horgan, 2020). Vulnerability is mainly related to the length of time that a crop is exposed to herbivores before it is harvested. For example, a long duration cereal variety will be more vulnerable to herbivores than a short duration variety (Horgan and Crisol, 2013). Each of these three factors will also interact, for example, a long duration variety might be more vulnerable to damage, but because it can compensate for damage over a longer time period, it may also be more tolerant of damage than a short duration variety. Understanding the relative contributions of each of these factors to observed damage to field crops will help select and optimally manage crops for low herbivore-induced yield losses. Despite this, few studies have addressed tolerance to stem borers (but see Rubia et al., 1989, 1996, 1997; Lv et al., 2008; Horgan et al., 2016), and fewer still have considered vulnerability to stem borers (but see Horgan and Crisol, 2013) during the selection of rice varieties for high yields in regions that are prone to stem borer attacks.

**Table 1:** Categories of herbivore-plant interactions and their definitions as related to this study.

Functional category	Definition	Mechanisms
Susceptibility	The relative favourability/suitability of a plant as food/refuge for herbivore attack and development	Linked to plant anatomical, mechanical, and biochemical properties
Vulnerability^a^	The relative exposure of a plant to herbivore attack and to the accumulation of damage	Linked to plant phenology and life duration
Resistance	The relative capacity of a plant to deter an herbivore attack (antixenosis) or reduce the fitness of an herbivore (antibiosis), compared to a susceptible variety	Linked to plant anatomical, mechanical, and biochemical properties
Tolerance	The relative capacity of a plant to withstand an herbivore attack and to continue to grow and develop in the face of damage or to compensate for the damage, thereby gaining a relative high biomass or greater reproductive output (e.g., cereal yield), relative to a non-tolerant plant under equal herbivore pressures	Linked to plant module size and growth rates

a Vulnerability may be regarded as a subcategory of susceptibility.

This study examines a range of rice varieties to assess the relative contributions of resistance, tolerance and vulnerability to observed levels of field damage from two stem borer species. Furthermore, the study assessed changes in the nature of rice-herbivore interactions as rice plants grow and develop. The main objective of the study was to determine the best phenotyping methods to distinguish categories of stem borer-rice interaction. In particular, by phenotyping for stem borer damage to long duration (≥125 days) and short duration (≤120 days) rice varieties under controlled conditions and under field conditions, we wished to assess the significance of vulnerability to field damage based on crop duration-. Screening for rice stem borer ‘resistance’ at national and international (e.g., CGIAR) institutes has been influenced by rapid assessment methods such as the standard evaluation system (SES) that grades damage to rice seedlings or young, tillering plants in stem borer-infested screen cages (Heinrichs. 1985; Togola et al., 2011; Roland et al., 2019). To date, most rice phenotyping studies have focused on only one stemborer species (i.e., 74% of 207 papers, usually with *Chilo suppressalis*: Horgan, unpublished). Furthermore, during phenotyping studies, researchers seldom examine plant ontogenetic effects on stem borer fitness (i.e., 23% of 207 studies investigated ontogeny by looking at interactions between stem borers and rice at different stages of plant development). Because stem borers occur in multi-species assemblages throughout most of their geographical distribution (Beuzelin et al., 2011; Sama et al., 2015; Sarwar, 2012; Horgan et al., 2016), we further set out to examine how well tests conducted using a single stem borer species at specific rice growth stages (either vegetative or reproductive) may be projected to a different stem borer species, and to field grown rice under varying levels of nitrogen fertilizer, and therefore exposed to varying intensities of stem borer attack throughout plant development. Based on our results, we make recommendations to improve phenotyping methods that evaluate the relative responses of rice varieties to stem borer assemblages.

## 2 Methods

### 2.1 Study species

We used two stem borer species - *Scirpophaga incertulas* (yellow stem borer: YSB) and *C. suppressalis* (striped stem borer: SSB) in our experiments. We used these two species because these were the most common species in the geographical region (Luzon Island, Philippines) where we conducted our experiments. In field experiments, a further species *Sesamia inferens* (pink stem borer: PSB) was observed, but at relatively low numbers. For experiments under controlled conditions, we collected adult YSB from farmers’ fields in Laguna, Philippines. Adult SSB were collected from the International Rice Research Institute (IRRI) experimental field station at Los Baños, Philippines. The adults were collected on the night prior to the experiments using entomological nets over planted rice fields. The moths were returned to an insectary (at 25-27 °C) and held overnight in acetate cages (50 × 60 × 80 cm: L × W × H) with rice plants (one species per cage, males and females together) until they were used in the experiments. To acquire larvae for experiments, we allowed adults to oviposit on rice (variety TN1) in large cages (120 × 80 × 90 cm; L × W × H). The egg masses were then collected and maintained in individual glass vials until neonates emerged. Neonates were used in experiments within 1 h of egg hatch.

### 2.2 Plant materials

We initially used ten rice lines with varying levels of resistance as determined from preliminary screening tests at IRRI. These were Taitung 16 (T16), IR36, IR40, IR50, IR62, IR66, and IR72, all of which reach maturity in <120 days, and IR68, IR70 and TKM6 that reach maturity in 125+ days. For a greenhouse experiment, we added two further lines, *Oryza rufipogon* (125 days to maturity) and IR64 (117 days to maturity), based on recent interest in the potential for wild rice species as resistance donors in breeding programs with IR64 as a recurrent parent (Reuolin et al., 2019; Sarangi and Islam, 2019). We did not use any recently released varieties because of generally insufficient supporting information regarding variety-stem borer responses (see above).

Seed was acquired through the IRRI Germplasm Bank with IR varieties acquired through the Plant Breeding, Genetics and Biotechnology Division of IRRI. The seed was initially sown to seedboxes with fine soil and transplanted at 9 days after sowing (DAS) to pots of paddy soil in a greenhouse or directly to soil in a screen house. The screenhouse had a series of concrete bays (2 × 20 m; W × L) that were open to the ground/soil. The bays were filled with paddy soil (approx. 60 cm deep) from the field station (Fig. S1). The whole facility was covered by 1 mm mesh that restricted the movement of arthropods, but allowed air circulation thereby maintaining cooler ambient temperatures (26-35 °C) than in the greenhouse (27-38 °C) (see also Crisol et al., 2013).

### 2.3 No-choice oviposition experiment

Seedlings of ten varieties were transplanted directly to paddy soil in the screen house facility, simulating field environments in terms of plant spacing and growing conditions (i.e., temperatures, humidity and light). The concrete bays were continuously flooded during the experiments (water depth = 5–20 cm) (Fig. S1). Seedlings of each variety (15 DAS) were planted in rows as three hills with 25 × 25 cm spacing. The rows were covered with fine mesh cages of dimensions 1.0 × 0.5 × 1.4 m (L × W × H), each containing a single row of three plants (of the same variety) (Fig. S1). The entire experiment consisted of 120 cages (10 varieties × 2 moth species × 6 replicates) set up as a completely randomized design. At 35 DAS, when plants were at the early tillering stage, each cage was infested with two gravid female YSB or SSB (ages unknown). We used plants of 35 DAS based on observations that plants of this age were suitable for larval development while at the same time being large enough to survive relatively heavy stem borer infestations. After 5 days, the cages and adults were removed and the plants were searched for egg masses. The numbers of egg masses per hill and on the cage structures (i.e., frame, screen, etc.) were noted and the masses collected and individually stored in scintillation vials to allow larvae to emerge. The numbers of larvae emerging from each egg mass were recorded.

### 2.4 Choice oviposition experiments

Oviposition choice experiments were conducted during the wet (YSB and SSB) and dry season (SSB only). The same protocol was followed in each season. Seedlings of each variety were transplanted at one seedling per hill to microplots in the screen house facility (described above). Each microplot consisted of 30 hills (10 varieties × 3 hills) distributed as 5 rows with a spacing of 35 × 35 cm between hills. The varieties were randomized within each microplot. Fertilizer was applied to the plots at a rate of 60 kg of Nitrogen/hectare ten days after transplanting (DAT). The microplots were individually covered with mesh cages of dimensions 2.45 × 2.10 × 1.4 m (L × W × H) (Fig. S1).

The entire experiment consisted of 12 or six cages (2 moth species × 6 replicates in the wet season, 1 species × 6 replicates in the dry season). In the experiment with 12 cages, the moth species were randomized across the cages with one species per cage. At 35 DAS the cages were each infested with 25 gravid female YSB or SSB. After 5 days, the cages and adults were removed and the plants and cages were searched for egg masses. The number of egg masses per hill was noted and the masses collected and individually stored in scintillation vials to allow larvae to emerge. The numbers of larvae emerging from each egg mass were recorded.

### 2.5 Stem borer performance on rice at vegetative and reproductive stages

Seed of each of 12 rice lines were sown to seedboxes with fine soil at two staggered intervals (T1 and T1+20 days) such that rice plants of 40 and 60 DAS were available at the same time. This allowed us to infest plants of the same variety at the vegetative stage (40 DAS) and at the reproductive stage (booting ≈ 60 DAS) at about the same time (i.e., larval development on vegetative and reproductive stages of each variety overlapped during some time). After nine days (9 DAS), seedlings from each group were transplanted to #6 pots (dimensions = 15.0 × 15.0 × 9.5 cm; height × rim diameter × base diameter) with 1.2 Kg of soil at one seedling per pot. The equivalent of 150 Kg Nitrogen/hectare was applied to the pots with applications split between basal and at 30 DAS.

At 35 DAS, the pots were covered with acetate cages (dimensions = 122.5 × 11.5 cm; height × diameter). Each cage had a fine mesh window and top to allow air to circulate. The pots were each infested with six neonates of either YSB or SSB using a paintbrush and passing the neonates through a slit in the acetate. Plants at the vegetative stage were infested at 40 DAS (early tillering). Because development times varied between rice varieties, the remaining plants were monitored until 60% of tillers were at the booting stage, this delayed infestations to some of the plants (TKM6, IR68, *O. rufipogon*) from 5 to 10 days. Reproductive plants were then infested (as indicated above) with six neonates of either moth species depending on the treatment. The entire experiment consisted of 288 plants (2 species × 12 varieties × 2 growth stages × 6 replicates). A further 144 pots were maintained with control, non-infested plants (72 sown at T1 and another 72 sown at T1+20 days – 6 per rice line at each time point). The experiment was set up as a completely randomized design.

Beginning two weeks after the infestations, the plants were daily monitored for emerging adults. Adults were collected on the day of emergence and were immediately frozen at −20 °C. After adults had stopped emerging, the plants were allowed to develop until they were ready for harvest (≥80% grain filled). The plants, including the control-non infested plants, were then destructively harvested noting the number of healthy and dead tillers. Plants were dried in a forced draught oven for two weeks and were then separated into shoots and panicles. The numbers of panicles were recorded and the grain recorded as filled or unfilled (number and dry weight). Moths were also dried and weighed, noting the sex and recording the date of emergence.

### 2.6 Field experiments

Field experiments were conducted during two seasons (2.6.1 and 2.6.2 below). During the first, wet season experiment, the two highest-damaged and two least-damaged rice lines were selected from ten test varieties. These were then examined in further detail during the second, dry season experiment to assess the repeatability of results and determine factors affecting damage levels under different nitrogen regimes. The main focus of the second experiment was to assess whether the rankings of least-damaged to highest-damaged varieties from the first experiment were maintained between seasons, and whether the results might be influenced by nitrogen levels.

#### 2.6.1 Field screening of ten varieties

Field plots of 5 × 5 m were planted with seedlings of one of each of the ten rice varieties during the wet season. The field site at IRRI had deep clay soils with about 4% organic matter. The plots were planted as six replicated blocks (randomized block design) with five rice hills of the susceptible rice variety TN1 between adjacent blocks. Seed was initially sown to dry seedbeds and the seedlings transplanted as one plant per hill to the puddled field plots at 28 DAS. Hills were spaced at 25 × 25 cm (planting distance). Plots were flooded until pre-harvest and received fertilizer (ammonium sulphate) equivalent to 100 Kg of nitrogen per hectare as a basal treatment, at three weeks after transplanting, and again at panicle initiation. Solophos, muriate of potash and zinc were applied basally with the ammonium sulphate. Plots received no pesticide treatments.

The rice plots were examined at 15 DAT (early tillering) for stem borer egg masses by carefully examining each hill. Rice plots were sampled for stem borer damage (dead heart tillers and whitehead-panicles) at 30 DAT (mid-tillering), 60 DAT (booting) and prior to harvest (when grain was ≥80% mature). At each sampling point, a single randomly-selected rice hill was carefully pulled and stored in a plastic sheath. A single hill, without apparent damage (i.e., no evidence of tunnel entrances, no dead hearts or whitehead-panicles and no apparent damage from other insects), was also selected and sampled from each plot. The sampled plants were examined in the laboratory noting the number of healthy or damaged vegetative and reproductive tillers. After examination in the laboratory, the plants were separated into different parts (roots, shoots, and panicles) and dried at 60 °C in a forced draught oven until a stable weight. After drying, we recorded root and shoot biomass, panicle weight and the weight and number of rice grains (filled and unfilled) for both the randomly selected plants and the selected non-infested plants. The number of whiteheads and total number of hills in each plot were counted prior to harvest. Yield was estimated at harvest based on panicles collected from 5 × 5 hills per plot.

#### 2.6.2 Field screening under varying nitrogen

A second experiment was incorporated into the Rice Ecosystem Functions Platform or ‘EcoFun’ (Horgan et al., 2019) with the same soil type as in the previous experiment. The platform consisted of a randomized block design with split plots, with nitrogen randomized to the main plots and variety randomized to sub-plots. The blocks each measured 33 × 12.5 m (L × W). Separate sub-irrigation channels were installed around each plot. These were connected to the main field canals for irrigation and drainage but prevented leakage of nutrients between adjacent fields or between plots within each field. Field plots were treated with one of three nitrogen levels: these were zero-added nitrogen (0 kg N ha^−1^), 60 kg N ha^−1^ and 150 kg N ha^−1^. Applications were divided between four nitrogen (ammonium sulphate) top dressings (basal, mid-tillering, panicle initiation and at one week before flowering). Solophos, muriate of potash and zinc were applied basally with the ammonium sulphate. No pesticides were used in the fields at any time during the experiments.

During the dry season, the plots were planted with IR66 and IR74 that had low levels of damage (i.e., few dead hearts and whitehead-panicles) during the previous season (2.6.1), and two varieties displaying high damage, IR68 and IR70 (2.6.1). The varieties were planted to the 15 nitrogen-treated plots as variety sub-plots (each 8.25 × 12.5 m; Fig. S2). Seed was initially sown to dry seedbeds and the seedlings transplanted as one plant per hill to the puddled field plots at 28 DAS. Hills were spaced at 25 × 25 cm.

During rice development, the plots were sampled for adult stem borers using sweepnets. Each plot was sampled at 30 and 60 DAT with 10 passes of a standard entomological sweep net (diameter = 40 cm). All moths were stored in 70% ethanol for identification. Prior to harvest (grain ≥80% mature) a single rice hill was pulled from each sub-plot and placed in a plastic sheath. Sampled hills were examined in the laboratory as described above (section 2.6.1). At the time of harvest, each plot was assessed for the incidence of whiteheads by counting the number of whiteheads in plots of 15 × 20 rice hills. The plots were harvested to estimate rice yield by cutting and threshing the panicles from 5 × 10 rice plants in each plot. The grain was oven dried and weighed.

### 2.7 Data analyses

Results from screen house choice and no-choice oviposition experiments were analyzed separately for each stem borer species using general linear models (GLMs) with variety as the only factor. Results from choice tests were ranked because of non-independence of values. Two-factor GLMs (variety, plant stage at infestation) were used to analyze damage and stem borer performance in the greenhouse (i.e., dead hearts = variety + plant stage + interactions) and also used to approximate tolerance (determined as relative changes in plant component weights between infested and control plants) from the greenhouse study for each stem borer species. Three-factor GLMs (that included sex as a factor) were used to assess pupal weights in the same experiment. Further analyses included both species together (3-factor and 4-factor GLMs, for analyses without sex and with sex, respectively, i.e., pupal weight = species + variety + rice stage + interactions; and pupal weight = species + sex + variety + rice stage + interactions). We conducted a range of Spearman's correlations between the results from the screen house (average egg masses per variety) and greenhouse studies (average dead tillers and average insect biomass at vegetative and reproductive stages) with average damage in the field experiments to assess the value of experiments from controlled environments in predicting field damage.

Data (plant traits, proportion of tillers that were dead, and proportion of panicles that were whiteheads) from the wet-season field trial were analyzed using repeated measures GLM with sampling date as the repeated measure and variety as the main factor. We conducted multiple regression with backward elimination to assess which rice traits were the best predictors of stem borer damage and fitness from the same trial. The plant trait data were from plants that were destructively harvested, but without apparent insect attack (i.e., parameters were estimated from attack-free plants, see above). The dry-season field trials were analyzed using a split-plot GLM with nitrogen level as the main plot and variety as the split plot.

Residuals were plotted after all parametric analyses and, where necessary were transformed to ensure homogeneity of variance. Transformations are indicated with the results. Statistical analyses were conducted using SPSS version 23.0 (IBM SPSS Statistics).

## 3 Results

### 3.1 Oviposition on ten varieties in the screen house

In the no choice experiments, females of both species deposited similar numbers of egg masses across rice varieties (Table 2). Whereas YSB demonstrated a preference for IR66 plants and avoided T16 plants (Table 2), SSB showed no significant preferences across varieties (wet season: Table 2; dry season: F_9,50_ = 0.643, P > 0.05, see Table S1). Similar results were noted when the numbers of larvae that emerged from the egg masses were analyzed (Table 2, Table S1).

**Table 2:** Oviposition by SSB and YSB on ten rice varieties in screen house choice and no-choice experiments during the wet season.^a^

Insect	Variety	Choice experiments^b^				No choice experiments^b^		
		Number of egg masses per plant	Proportion of egg masses on each plant	Number of larvae emerged on each plant	Proportion of larvae on each plant	Number of egg masses per plant	Number of egg masses per cage^c^	Number of larvae emerged on each plant
SSB								
	IR36	0.33 (0.21)	0.11 (0.07)	32.17 (30.21)	0.15 (0.14)	0.17 (0.17)	0.17 (0.17)	9.17 (9.17)
	IR40	0.17 (0.17)	0.06 (0.06)	1.67 (1.67)	0.01 (0.01)	0.00 (0.00)	0.00 (0.00)	0.00 (0.00)
	IR50	0.17 (0.17)	0.17 (0.17)	10.83 (10.83)	0.17 (0.17)	0.00 (0.00)	0.00 (0.00)	0.00 (0.00)
	IR62	0.00 (0.00)	0.00 (0.00)	0.00 (0.00)	0.00 (0.00)	0.00 (0.00)	0.00 (0.00)	0.00 (0.00)
	IR66	0.17 (0.17)	0.06 (0.06)	1.67 (1.67)	0.03 (0.03)	0.33 (0.21)	0.33 (0.21)	14.50 (14.50)
	IR68	0.22 (0.21)	0.11 (0.07)	21.17 (19.23)	0.09 (0.07)	0.17 (0.17)	0.17 (0.17)	19.17 (19.17)
	IR70	0.00 (0.00)	0.00 (0.00)	0.00 (0.00)	0.00 (0.00)	0.00 (0.00)	0.00 (0.00)	0.00 (0.00)
	IR72	0.33 (0.33)	0.11 (0.07)	14.17 (9.36)	0.18 (0.15)	0.17 (0.17)	0.17 (0.17)	17.33 (17.33)
	Taitung 16	0.33 (0.21)	0.22 (0.16)	30.33 (28.38)	0.52 (0.41)	0.00 (0.00)	0.00 (0.00)	0.00 (0.00)
	TKM6	0.17 (0.17)	0.17 (0.17)	1.17 (1.17)	0.17 (0.17)	0.00 (0.00)	0.00 (0.00)	0.00 (0.00)
F-variety		0.643ns	0.643ns	0.719ns	0.719ns	1.087ns	1.087ns	0.667ns
YSB								
	IR36	1.00 (0.26)ab	0.19 (0.06)ab	26.00 (12.61)ab	0.32 (0.16)ab	0.17 (0.17)	0.83 (0.40)	12.83 (12.83)
	IR40	0.33 (0.21)ab	0.04 (0.02)ab	12.00 (8.77)ab	0.04 (0.03)ab	0.33 (0.21)	0.33 (0.21)	1.83 (1.83)
	IR50	1.17 (0.54)ab	0.15 (0.07)ab	24.17 (11.29)ab	0.19 (0.11)ab	0.00 (0.00)	0.67 (0.67)	0.67 (0.67)
	IR62	0.67 (0.33)ab	0.09 (0.04)ab	21.83 (11.23)ab	0.14 (0.06)ab	0.33 (0.33)	1.17 (0.54)	8.50 (8.50)
	IR66	1.33 (0.21)b	0.23 (0.05)b	33.67 (14.02)b	0.47 (0.29)b	0.17 (0.17)	0.17 (0.17)	2.00 (2.00)
	IR68	0.50 (0.34)ab	0.05 (0.03)ab	18.83 (17.47)ab	0.09 (0.08)ab	0.67 (0.21)	1.00 (0.37)	28.33 (17.63)
	IR70	0.17 (0.17)ab	0.03 (0.03)ab	4.50 (4.50)ab	0.03 (0.03)ab	0.17 (0.17)	0.33 (0.33)	1.83 (1.83)
	IR72	0.17 (0.17)ab	0.02 (0.02)ab	3.17 (3.17)ab	0.02 (0.02)ab	0.33 (0.21)	0.83 (0.40)	6.50 (6.50)
	Taitung 16	0.00 (0.00)a	0.00 (0.00)a	0.00 (0.00)a	0.00 (0.00)a	0.33 (0.21)	0.50 (0.22)	13.50 (13.50)
	TKM6	1.33 (.61)ab	0.19 (0.08)ab	49.17 (26.52)ab	0.50 (0.29)ab	0.33 (0.21)	0.67 (0.42)	18.00 (17.02)
F-variety		3.276**	3.276**	2.893*	2.925*	0.893ns	0.708ns	0.474ns

a Results for dry season experiment with SSB are presented in Table S1

b Results from GLM for F_9,50,_ data was ranked before analyses. ** = P ≤ 0.01; * = P ≤ 0.05; ns = P ≥ 0.05, lowercase letters indicate homogenous groups (Tukey test, P < 0.05). Numbers in parentheses are standard errors.

c Numbers of egg masses per cage also includes egg masses that were deposited on the cage structures (i.e., cage frame, mesh, etc.).

There were no significant correlations between the results from choice and no-choice experiments (YSB: Rs_10_ = −0.350, P = 0.322; SSB wet season: Rs_10_ = −0.462, P = 0.179; SSB dry season: Rs_10_ = −0.588, P = 0.094). There were no significant correlations between egg-laying by YSB and SSB or the number of larvae of each species emerging from egg masses collected on the rice varieties (YSB and SSB wet season: egg masses: Rs_10_ = −0.103, P = 0.777; emerged larvae: Rs_10_ = 0.494, P = 0.147; YSB wet season, SSB dry season: egg masses: Rs_10_ = 0.485, P = 0.156; emerged larvae: Rs_10_ = −0.296, P = 0.407). There was also no correlation between results from choice oviposition experiments with SSB in the wet season and the dry season (egg masses: Rs_10_ = −0.592; P = 0.072; proportion of masses per plant Rs_10_ = −0.222; P = 0.538).

We found no relation between the numbers or proportions (from the total per cage) of YSB egg masses per variety and either plant biomass or tiller number (0.153 ≥ Rs ≤ 0.386). The numbers/proportions of SSB egg masses were negatively correlated with tiller number in the dry-season experiment (masses Rs_10_ = −0.655, P = 0.040; proportions Rs_10_ = −0.650, P = 0.042), but not with plant biomass (masses Rs_10_ = −0.013, P = 0.957; proportions Rs_10_ = −0.483, P = 0.157). No other correlations were significant.

### 3.2 Performance on plants at different growth stages in the greenhouse

Larvae of both species had greater survival, shorter development times, heavier adults, achieved a greater total biomass, and caused greater tiller damage (i.e., proportion of tillers dead) when attacking plants at the vegetative stage compared to the reproductive stage (Table 3). Development times were longer and adults lighter for males of both species. Damage to tillers and stem borer fitness varied across rice lines (Table 3); in general, SSB performed well and resulted in highest fitness and a higher proportion of dead hearts on TKM6; insect biomass was generally low on *O. rufipogon* and T16 (Table 3). The proportion of tillers that were dead (dead hearts) due to YSB was also highest on TKM6 and relatively low on IR36, IR40, IR50 and IR66; however, YSB on these latter plants often had heavy pupae and rapid development (Table 3). In contrast to the generally lower SSB fitness on *O. rufipogon* and T16, the proportion of tillers that were dead was generally high because of low tillering in these two lines during the experiment (Table 3). Several of the interaction terms were significant: increasing SSB fitness on T16 plants, but declining fitness on IR62 as these plants matured, produced a significant [variety × plant stage] interaction (Table 3); significant two- and three-way interactions with sex were due to often large and rapidly developing SSB males on IR36 and *O. rufipogon* at the vegetative stages but small females on these same lines at the reproductive stages (Table 3). Similarly, a decline in the proportion of dead tillers on mature *O. rufipogon* plants infested with YSB relative to vegetative plants produced a significant [variety × plant stage] interaction for dead tillers (Table 3). Significant two- and three-way interactions with sex in YSB were mainly due to relatively low weights of female pupae on mature IR62 and *O. rufipogon* (Table 3).

**Table 3:** Performance by stem borer larvae on 12 rice lines in a greenhouse experiment. Plants were infested at the vegetative (40 DAS) and reproductive (booting) stages with larvae of either SSB or YSB.

Species	stage	Variety or wild rice species	Tiller number^a^	Dead heart and white head (%)^a^	Survival to adult^a^	Development time (male)(days) a	Development time (female)(days) a	Adult weight (male)(mg) a	Adult weight (female)(mg) a	Insect biomass (mg) a
SSB										
	Vegetative									
		IR36	13.83 (0.48)gh	19.35 (1.62)a	0.40 (0.04)ab	34.14 (0.79)	32.53 (1.35)bcd	11.86 (0.83)	24.09 (1.89)ab	70.14 (9.64)ab
		IR40	15.33 (1.20)h	25.10 (3.10)ab	0.32 (0.07)ab	34.17 (1.00)	31.07 (0.26)ab	8.50 (0.76)	23.35 (1.57)a	50.41 (10.04)ab
		IR50	12.67 (0.76)fgh	21.08 (3.29)a	0.32 (0.06)a	37.57 (1.08)	41.40 (0.99)d	8.71 (0.25)	21.08 (2.09)a	48.25 (9.19)a
		IR62	11.33 (0.61)defg	29.19 (2.83)ab	0.48 (0.05)ab	34.59 (0.81)	32.25 (0.65)abc	8.69 (0.39)	22.11 (1.01)a	95.37 (16.38)ab
		IR64	9.50 (0.92)bcde	26.27 (4.37)ab	0.35 (0.03)ab	35.60 (1.80)	33.10 (0.88)abc	8.72 (0.52)	21.01 (1.39)ab	54.30 (5.85)ab
		IR66	10.00 (1.03)cdefg	22.09 (3.04)ab	0.40 (0.04)ab	34.64 (0.34)	33.87 (0.99)bcd	12.49 (0.77)	23.28 (0.98)ab	72.00 (5.60)ab
		IR68	9.17 (0.79)abcd	24 .43 (2.99)ab	0.42 (0.07)ab	34.84 (2.21)	35.04 (0.81)abc	9.01 (0.62)	20.13 (2.92)ab	58.49 (9.84)ab
		IR70	11.17 (0.70)cdef	26.95 (2.77)ab	0.38 (0.06)ab	33.33 (0.56)	30.17 (0.53)bcd	8.99 (1.14)	21.75 (3.01)ab	57.33 (7.07)ab
		IR72	11.67 (0.21)efgh	17.17 (0.32)ab	0.30 (0.04)ab	33.22 (0.79)	33.08 (1.49)abc	10.23 (0.44)	23.55 (1.92)ab	40.53 (7.67)ab
		*O. rufipogon*	5.50 (0.43)a	16.94 (6.27)ab	0.33 (0.05)ab	38.29 (2.90)	32.14 (1.42)cd	11.59 (0.96)	18.01 (1.88)a	54.04 (11.35)a
		Taitung 16	6.50 (0.18)ab	23.21 (2.91)ab	0.25 (0.03)ab	42.67 (1.72)	22.61 (5.03)bcd	9.16 (0.23)	12.63 (2.42)a	28.64 (7.29)a
		TKM6	8.83 (0.79)abc	25.88 (5.34)b	0.50 (0.05)b	33.35 (1.28)	31.00 (2.40)a	10.63 (1.10)	24.91 (2.11)b	90.25 (12.04)b
	Reproductive									
		IR36	10.00 (0.68)	4.86 (3.12)	0.20 (0.09)	49.89 (3.90)	43.24 (1.93)	5.89 (0.15)	13.45 (1.45)	17.26 (7.80)
		IR40	10.17 (0.75)	7.24 (3.28)	0.42 (0.05)	40.60 (2.26)	36.79 (1.40)	8.31 (0.77)	11.63 (1.53)	40.67 (6.03)
		IR50	10.00 (0.45)	2.08 (2.08)	0.15 (0.06)	50.00 (2.58)	48.31 (2.22)	6.64 (1.03)	13.02 (0.69)	14.40 (6.68)
		IR62	9.33 (0.67)	9.31 (4.59)	0.17 (0.04)	44.00 (1.55)	40.50 (1.62)	7.19 (0.81)	13.72 (0.39)	13.97 (3.66)
		IR64	8.00 (0.45)	10.83 (4.90)	0.37 (0.06)	40.13 (3.68)	41.35 (2.54)	9.18 (0.82)	20.70 (2.25)	47.99 (8.45)
		IR66	9.60 (0.61)	2.50 (2.04)	0.36 (0.09)	39.95 (1.59)	50.31 (4.55)	7.95 (1.11)	11.36 (1.46)	40.48 (11.58)
		IR68	7.17 (0.48)	17.68 (6.64)	0.25 (0.05)	48.69 (4.29)	36.77 (1.38)	9.39 (0.69)	15.81 (2.53)	34.60 (8.92)
		IR70	7.00 (0.58)	18.81 (7.08)	0.45 (0.03)	49.39 (3.00)	47.71 (2.72)	7.35 (0.84)	14.65 (2.04)	47.96 (6.17)
		IR72	9.33 (1.20)	9.03 (6.31)	0.47 (0.04)	44.60 (2.74)	40.40 (2.75)	8.95 (0.84)	16.91 (1.35)	63.05 (9.85)
		*O. rufipogon*	7.17 (0.60)	11.27 (5.64)	0.28 (0.03)	50.22 (5.43)	49.50 (5.00)	7.98 (0.84)	13.64 (1.89)	31.94 (4.82)
		Taitung 16	6.50 (0.34)	6.67 (4.22)	0.40 (0.07)	47.70 (1.97)	48.18 (3.06)	8.41 (0.58)	17.18 (1.50)	49.07 (10.36)
		TKM6	6.50 (0.62)	27.10 (5.27)	0.40 (0.07)	38.08 (1.85)	34.15 (1.17)	9.69 (1.12)	20.91 (3.44)	57.85 (13.18)
	F-Variety^b^ (V)		17.349***	2.568**	2.158*		6.089***		2.863***	2.524**
	F-Plant stage^c^ (P)		52.430***	52.617***	4.203*		264.526***		84.591***	30.395***
	F-Sex^d^ (S)						19.078***		482.746***	
	V × P^b^		3.643***	1.253ns	3.772***		3.019***		4.417***	4.318***
	V × S^2^						2.248**		2.081*	
	P × S						1.170ns		26.652***	
	V × P × S^2^						3.307***		1.992*	
YSB										
	Vegetative									
		IR36	13.17 (1.08)f	34.00 (10.55)a	0.33 (0.08)	36.73 (0.99)	34.50 (0.75)ab	5.17 (0.26)	15.66 (2.01)ab	37.58 (12.04)ab
		IR40	13.17 (0.40)def	28.75 (4.68)a	0.33 (0.04)	38.00 (1.84)	34.54 (0.40)ab	5.96 (0.38)	18.07 (1.81)bc	37.60 (5.36)ab
		IR50	13.60 (0.49)ef	40.46 (5.39)a	0.30 (0.03)	40.00 (1.60)	35.63 (0.77)abcd	7.24 (0.71)	14.11 (1.56)bc	35.43 (6.37)ab
		IR62	9.00 (1.44)cd	39.26 (3.52)ab	0.42 (0.04)	36.81 (0.48)	37.03 (0.80)abc	5.93 (0.31)	17.93 (1.91)ab	45.72 (5.77)ab
		IR64	8.83 (0.79)bc	45.37 (6.28)ab	0.33 (0.09)	37.28 (1.27)	37.22 (1.10)abc	6.21 (0.23)	16.24 (1.64)bc	44.06 (10.72)b
		IR66	10.56 (0.76)cde	28.65 (2.73)a	0.26 (0.03)	39.33 (1.25)	39.19 (2.15)abcd	5.64 (0.29)	16.29 (0.93)bc	26.95 (6.03)ab
		IR68	9.60 (0.66)bc	30.54 (5.79)ab	0.28 (0.06)	40.50 (1.02)	39.17 (1.87)bcd	7.05 (0.16)	18.43 (1.02)c	37.17 (5.73)ab
		IR70	11.79 (0.31)cdef	28.30 (4.60)ab	0.24 (0.03)	41.75 (1.43)	38.00 (0.52)abcd	5.97 (0.06)	15.33 (1.12)bc	32.29 (7.82)ab
		IR72	10.40 (0.42)cde	37.79 (5.83)a	0.20 (0.03)	40.10 (0.78)	36.50 (0.65)abcd	5.21 (0.10)	20.17 (1.45)bc	32.71 (6.98)ab
		*O. rufipogon*	6.50 (0.67)ab	54.35 (4.67)ab	0.33 (0.08)	39.43 (2.31)	39.17 (2.42)cd	4.95 (0.41)	11.42 (1.27)a	32.86 (11.77)ab
		Taitung 16	6.33 (0.21)a	47.62 (5.86)ab	0.27 (0.06)	43.10 (2.16)	42.86 (2.50)d	5.45 (0.20)	14.77 (0.93)ab	25.50 (5.29)a
		TKM6	7.83 (0.65)ab	56.06 (10.78)b	0.25 (0.04)	39.10 (1.43)	34.90 (0.73)a	6.14 (0.39)	19.96 (1.15)bc	29.03 (7.70)ab
	Reproductive									
		IR36	10.80 (0.31)	23.29 (4.26)	0.20 (0.04)	39.70 (0.91)	37.92 (3.69)	4.74 (0.43)	11.79 (1.18)	14.50 (4.38)
		IR40	9.33 (0.33)	33.89 (10.09)	0.27 (0.07)	38.50 (2.23)	37.21 (2.12)	5.96 (0.25)	11.90 (1.19)	25.09 (5.44)
		IR50	10.00 (0.37)	24.92 (6.10)	0.35 (0.06)	43.96 (3.15)	35.90 (0.45)	5.77 (0.26)	14.24 (0.89)	36.10 (4.17)
		IR62	10.17 (0.75)	34.25 (6.89)	0.20 (0.07)	41.28 (4.94)	39.90 (1.45)	6.06 (0.11)	8.56 (0.89)	13.93 (4.50)
		IR64	7.83 (0.48)	34.84 (5.90)	0.35 (0.08)	43.95 (4.91)	36.22 (0.71)	5.55 (0.37)	13.41 (0.96)	37.53 (6.13)
		IR66	9.00 (0.73)	15.00 (5.44)	0.32 (0.05)	39.43 (0.53)	38.00 (1.48)	7.07 (0.45)	11.86 (1.71)	32.09 (5.65)
		IR68	7.20 (0.65)	47.50 (7.73)	0.24 (0.07)	44.33 (1.63)	41.33 (1.25)	6.28 (0.08)	19.70 (1.96)	34.07 (10.23)
		IR70	8.00 (0.73)	43.67 (7.75)	0.24 (0.03)	40.60 (0.76)	37.88 (1.27)	5.56 (0.60)	17.36 (1.63)	30.01 (4.67)
		IR72	9.00 (0.86)	20.63 (6.70)	0.22 (0.03)	37.25 (1.45)	45.67 (3.83)	5.53 (0.58)	17.34 (1.82)	23.42 (2.04)
		*O. rufipogon*	6.67 (0.49)	23.06 (5.62)	0.15 (0.04)	45.31 (2.39)	44.00 (1.29)	5.00 (0.47)	8.29 (0.26)	10.69 (3.23)
		Taitung 16	5.17 (0.31)	27.22 (14.92)	0.12 (0.05)	39.22 (0.40)	47.75 (1.17)	6.02 (0.12)	13.60 (1.36)	10.97 (5.26)
		TKM6	6.67 (0.21)	61.90 (14.14)	0.18 (0.03)	37.75 (2.46)	34.00 (0.52)	4.76 (0.19)	17.00 (1.77)	28.40 (6.03)
	F-Variety (V)^b^		18.771***	2.855***	1.513ns		4.792***		6.242***	1.997*
	F-Plant stage (P)^c^		43.028***	4.836*	6.865**		9.236***		24.913***	11.882***
	F-Sex (S)^d^						9.242***		769.235***	
	V × P^b^		2.815***	1.898*	1.455ns		1.002ns		1.623ns	1.517ns
	V × S^2^						2.085*		5.531***	
	P × S^2^						0.471ns		11.306***	
	V × P × S^2^						1.631ns		3.041***	

a Numbers in parentheses are standard errors; lowercase letters indicate homogenous variety/rice line groups for each stem borer species analyzed separately (Tukey, P ≤ 0.05); note that homogenous groups are calculated using the combined results for vegetative and reproductive plants and are indicated only once per variety/wild rice species for each stemborer species.

b DF = 11,120 for two factor tests, 11,240 for three factor tests (i.e., including the factor sex); ns = P > 0.05, * = P ≤ 0.05, ** = P ≤ 0.01, *** = P ≤ 0.005.

c DF = 1,120 for two factor tests, 1,240 for three factor tests (i.e., including the factor sex); * = P ≤ 0.05, ** = P ≤ 0.01, *** = P ≤ 0.005.

d DF = 1,240; *** = P ≤ 0.005.

Overall, the results indicated higher susceptibility and damage by stem borers to TKM6 and relatively low susceptibility and damage to T16 and *O. rufipogon* (combined YSB and SSB in analysis: dead tillers F_11,240_ = 4.619, P < 0.001; survival F_11,240_ = 1.141, P < 0.05; species total biomass F_11,240_ = 2.327, P < 0.01: Table 3). SSB had greater survival and attained a higher biomass than YSB, but caused relatively lower damage (combined data: dead tillers F_1,240_ = 114.160, P < 0.001; survival F_1,240_ = 26.369, P < 0.001; species total biomass F_1,240_ = 64.944, P < 0.001: Table 3). Dead tillers and stem borer survival were consistently higher at the vegetative stage (combined data: dead tillers F_1,240_ = 30.070, P < 0.001; survival F_1,240_ = 10.894, P < 0.001; species total biomass F_1,240_ = 43.003, P < 0.01: Table 3). A greater reduction in the fitness of SSB on mature plants relative to the reduction in YSB produced a significant [species × plant stage] interaction (F_11,240_ = 5.354, P < 0.05).

**Fig. 1:**
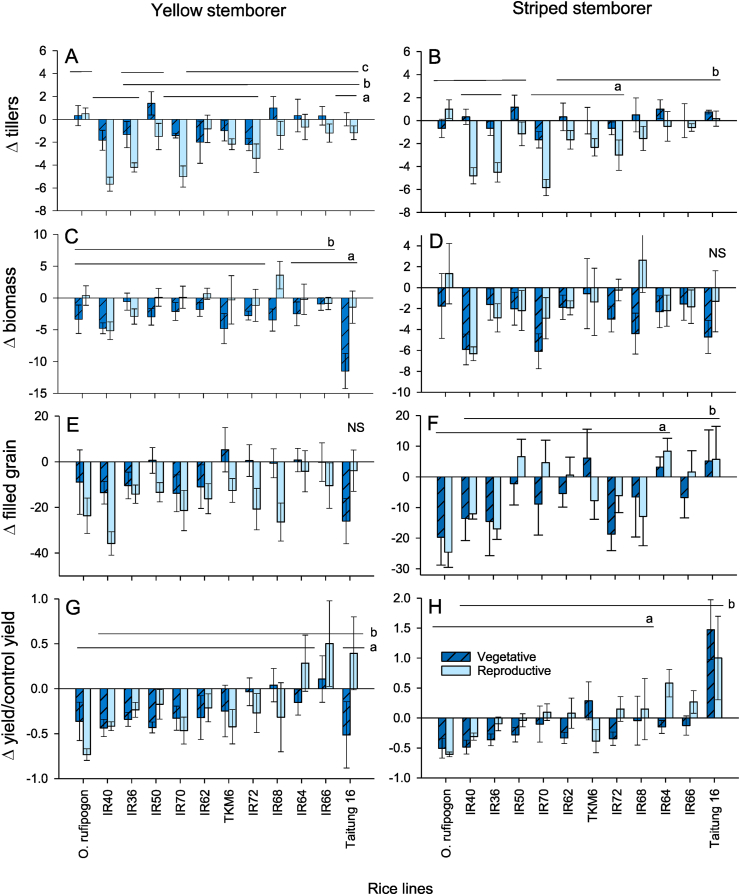
Differences between infested and non-infested, control plants (Δ) in the number of tillers (A,B), the above ground biomass (C.D), and the proportion of rice grain that was filled (E, F) for experiments with YSB (A, C, E) or SSB (B,D,F) in the greenhouse study. Proportional losses in yield (g dry weight) are also indicated for YSB (G) and SSB (H). Error bars are indicated; lowercase letters indicate homogenous rice line groups based on combined data from both plant stages and both stem borer species (Tukey > 0.05; N = 6). For further details see Table S2.

Compared to SSB, infestation by YSB resulted in greater losses to yield across varieties (proportion of grain that was filled: F_1,235_ = 7.952, P < 0.001; weight of filled grain: F_1,235_ = 5.506, P < 0.05; proportional change in yield: F_1,235_ = 7.447, P < 0.001); otherwise, damage (changes to tiller number, changes to biomass, and changes to panicle number) was similar between species (Fig. 1; Table S2). Damage differed across rice lines, with IR50, IR64, T68 and *O rufipogon* often compensating for damage to tillers and shoots (tiller number: F_11,235_ = 8.831, P < 0.001; shoot biomass: F_11,235_ = 2.433, P < 0.001) and IR64, IR66 and T16 compensating (often overcompensating) through increased yields (proportion of grain that was filled: F_11,235_ = 5.463, P < 0.001; weight of filled grain: F_11,235_ = 4.682, P < 0.001; proportional change in yield: F_11,235_ = 7.443, P < 0.001; Fig. 1; Table S2). The stage at which the plants were infested affected damage: plants infested at the booting stage had generally greater losses to tiller number but lost less shoot biomass compared to plants infested at 40 DAS (tiller number: F_1,235_ = 54.295, P < 0.001; biomass: F_1,235_ = 4.681, P < 0.001; Fig. 1A–D; Table S2). Infestation at the booting stage resulted in smaller losses to filled grain weight (F_1,235_ = 4.281, P < 0.05), but this did not significantly affect the relative losses to yield (Fig. 1G and H). Early attack (40 DAS) by SSB caused greater losses to yields than attacks by this species at the booting stage, but the opposite occurred with YSB (proportional change in yield, species × plant stage interaction: F_11,235_ = 8.645, P < 0.001: Fig. 1G and H, Table S2).

In general, losses to tillers, biomass and panicles were poorly correlated with insect fitness parameters (2/60 correlations at P < 0.05). Different estimates of losses to yield were often highly correlated (loss in grain number, proportional reduction in filled grain, loss in grain weight and proportional loss in yield [12/24 correlations at P < 0.05], particularly for plants infested at the reproductive stage [9/12 correlations at P < 0.05]). Insect fitness parameters were poorly correlated between moth species and between moths of the same species infesting plants at different growth stages (Table 4). Levels of damage from SSB and YSB were often well correlated for plants infested at the booting stage (7/8 correlations) but not at the vegetative stage (0/8 correlations: Table 4). Insect fitness parameters from the greenhouse were not correlated with preferences (egg-laying) from the screen house study.

**Table 4:** Spearman correlation coefficients [coefficient (P-value)] for insect fitness parameters and damage estimates from the greenhouse experiment. Note that variety ‘tolerance’ measured after infestation during the reproductive stage was generally consistent between stem borer species.

Parameters^1^	SSB different stages^2^	YSB different stages^2^	Vegetative different species^2^	Reproductive different species^2^
Survival	−0.362 (0.247)	0.136 (0.674)	0.029 (0.830)	−0.099 (0.760)
Insect weight	−0.308 (0.331)	0.154 (0.633)	0.112 (0.729)	0.063 (0.846)
Female weight	−0.140 (0.665)	0.587 (0.045)*	0.364 (0.245)	0.462 (0.131)
Female development time	0.130 (0.688)	−0.063 (0.846)	0.587 (0.045)*	0.228 (0.477)
Dead heart	0.483 (0.112)	−0.147 (0.649)	0.112 (0.729)	0.713 (0.009)**
Δ tillers	0.464 (0.129)	0.535 (0.073)	0.473 (0.121)	0.907 (0.001)***
Δ biomass	0.217 (0.499)	0.294 (0.354)	0.077 (0.812)	0.392 (0.208)
Δ panicles	0.615 (0.033)*	0.565 (0.055)*	0.448 (0.144)	0.731 (0.007)**
Δ total grain	0.608 (0.036)*	0.622 (0.031)*	0.126 (0.697)	0.643 (0.024)*
Δ prop grain filled	0.608 (0.036)*	0.266 (0.404)	0.287 (0.366)	0.650 (0.022)*
Δ grain weight	0.517 (0.085)	0.224 (0.484)	0.133 (0.681)	0.720 (0.008)**
Δ grain weight prop	0.484 (0.110)	0.492 (0.105)	0.226 (0.479)	0.760 (0.004)***

1: See Table S10 for calculation of differences in plant condition between control and infested plants (Δ) as related to tillers, biomass, panicles, etc.

2: * = P ≤ 0.05, ** = P ≤ 0.01, *** = P ≤ 0.001.

### 3.3 Field experiment with 10 varieties

**Fig. 2:**
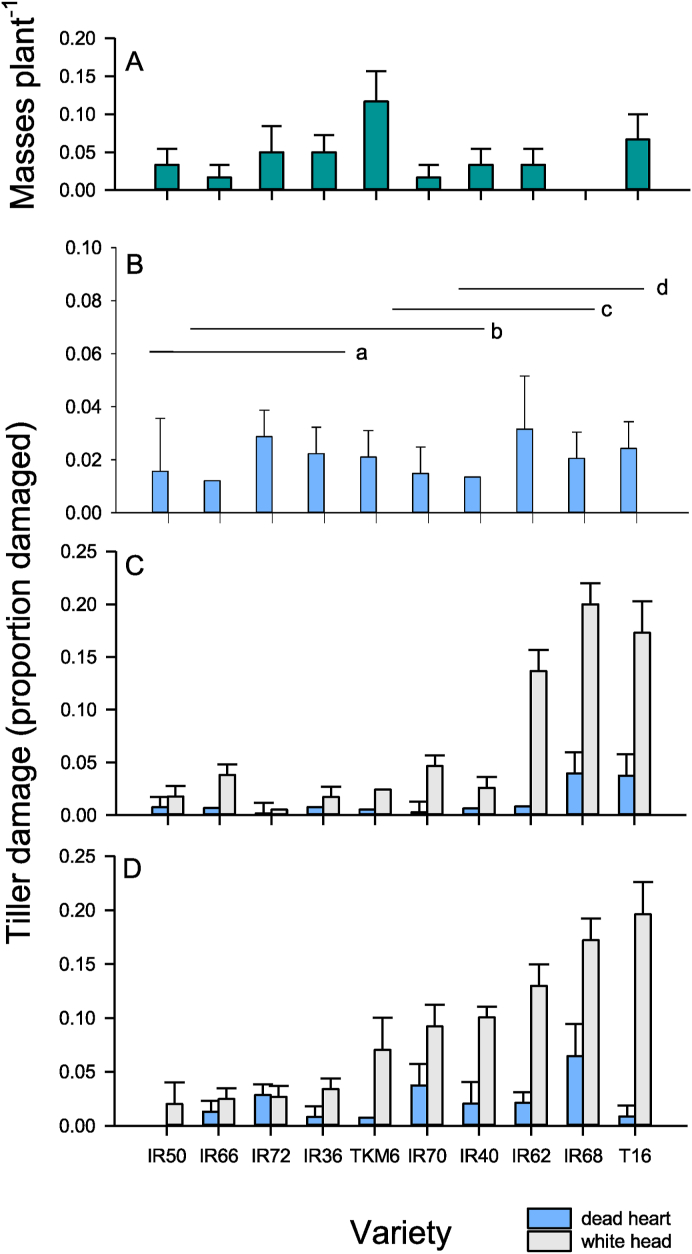
Results from field experiment with ten rice varieties. Data indicate (A) the number of YSB egg masses recorded in plots at 15 days after transplanting and tiller damage at (B) early, (C) mid, and (D) late season sampling. Standard errors are indicated. Homogenous variety groups are indicated based on Tukey tests (P > 0.05).

Stemborers were the most abundant lepidopterans in the field experiments. Plants were also damaged by leaf folders and other chewing caterpillars (15–49% of leaves having minor damage). There were no differences in the numbers of egg masses (all YSB) deposited on plants in the field experiment at the seedling stage (F_9,50_ = 1.265, P > 0.05: Fig. 2A). Damage increased over time (dead hearts: F_2,100_ = 8.386, P < 0.001; whiteheads = F_2,100_ = 5.038, P < 0.001); but only whitehead damage differed between varieties (dead heart: F_9,50_ = 1.078, P > 0.05; whitehead: F_9,50_ = 29.267, P < 0.001: Fig. 2B–D). Estimates of whitehead incidence from plants sampled at harvest time were highly correlated with estimates from whole plot counts (Rs_10_ = 0.967, P < 0.001). Damage to tillers was not correlated between 30 DAT and harvest samples (Rs_10_ = 0.248, P = 0.489), but was correlated between 60 DAT samples and harvest samples (Rs_10_ = 0.806, P = 0.005). Information on growth and yields of rice plants is presented in Table S3. Variety yields were not correlated with tiller damage during the experiment (Rho = −0.195).

The incidence of dead heart at 30 DAT was not related to the tiller number, tiller weight, shoot weight, frondiness (i.e., leaves per tiller and/or leaf weight) or plant height among healthy plants across varieties (the best predictor was based on a model that included the number of tillers per plant, the number of leaves per tiller and tiller weight: F_3,9_ = 14.177, P = 0.004)(Fig. S3). The tiller weight of healthy plants was the best predictor of whitehead damage across varieties at the time of harvest (F_1,8_ = 7.584, P = 0.028: Fig. S3). Results from the screen house oviposition experiment were not correlated with oviposition or damage in the field experiment (Table 5). The proportion of dead vegetative tillers in the greenhouse was correlated with field oviposition and dead hearts in the field; but results from greenhouse experiments with reproductive plant stages were not correlated with observed damage in the field (Table 5).

**Table 5:** Spearman correlation coefficients between YSB population and damage estimates from controlled (screen house and greenhouse) and non-controlled (field plots) experiments.

Controlled environment	Field parameters^a^		
	Oviposition	Vegetative dead heart	Reproductive white head
*Screen house*			
Choice oviposition YSB	−0.028 (0.939)	−0.293 (0.412)	−0.396 (0.257)
*Greenhouse*			
Vegetative Dead tillers YSB	0.772 (0.009)**	0.624 (0.054)*	0.139 (0.701)
Vegetative YSB biomass	−0.204 (0.572)	0.248 (0.572)	0.030 (0.934)
Reproductive Dead tillers YSB	−0.043 (0.906)	0.055 (0.881)	0.600 (0.067)
Reproductive YSB biomass	−0.611 (0.060)	−0.697 (0.025)*	−0.430 (0.214)

a * = P ≤ 0.05, ** = P ≤ 0.01.

### 3.4 Effects of nitrogen on stem borer damage

**Fig. 3:**
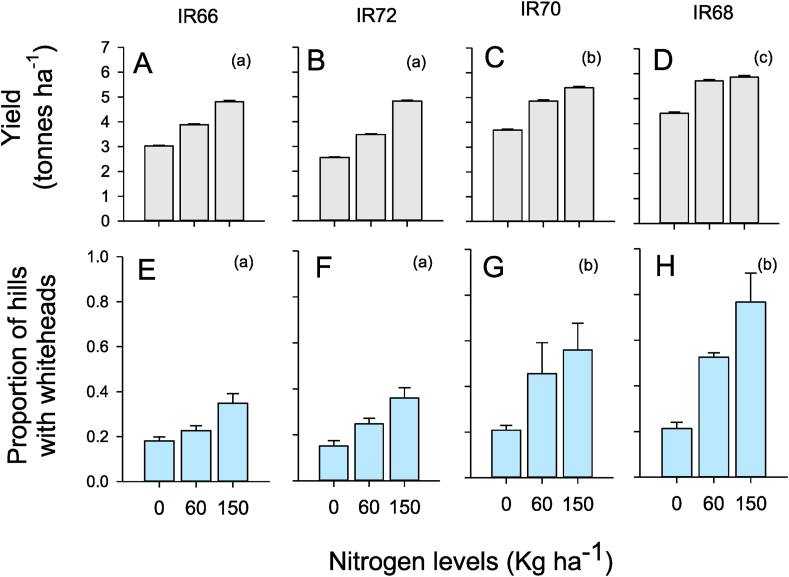
Yields of (A) IR66, (B) IR72, (C), IR70 and (D) IR68 in field plots treated with three levels of nitrogenous fertilizer. The proportion of whiteheads per hill is also indicated for (E) IR66, (F) IR72, (G) IR70 and (H) IR68. Field plots were treated with 0, 60 or 150 Kg of added nitrogen per hectare (N = 5). Standard errors are indicated and lowercase letters indicate homogenous variety groups (Tukey P > 0.05). See Table S4 for further details.

Adult YSB and PSB were captured in sweep nets in the dry-season field plots at 60 DAT. More adults were captured in the low nitrogen plots (F_2,59_ = 83.601, P < 0.001) and in plots of IR68 and IR70 (F_3,59_ = 1023.898, P < 0.001: Table S4); there was a significant interaction because no moths were captured in the high nitrogen plots (Table S4). Plants grown under high nitrogen were larger and taller with higher numbers of tillers (Table S4). Nitrogen increased the yield of each of the four varieties (F_2,59_ = 27.456, P < 0.001: Fig. 3A–D). The two high-damage varieties, IR68 and IR70, had the highest yields based on examination of whole plants (Table S4) and harvested plots (F_3,59_ = 134.345, P < 0.001, Fig. 3A–D). Nitrogen also increased the number of whiteheads per plant (Table S4) and per plot in all varieties (F_2,59_ = 99.489, P < 0.001, Fig. 3E–H). However, there were significant [variety × nitrogen] interactions because whitehead number was similar among the four varieties under low nitrogen, but increased on IR68 and IR70 at higher nitrogen rates (Fig. 3E–H, Table S4). Whiteheads per plant and per plot were correlated (Rs_12_ = 0.707, P = 0.010: Table S4). Total plant biomass (of non-damaged plants) was the best predictor of whitehead damage across nitrogen levels and varieties (R^2^ = 0.478; F_1,11_ = 9.612, P = 0.013). The ranking of the four varieties from least to most damaged (IR72 < IR 66 < IR70 < IR68) corresponded with the results from the wet season field experiment (Fig. 2, Fig. 3).

## 4 Discussion

Our results highlight some of the challenges (i.e., several stem borer species, complex categories of interaction, and major ontogenetic shifts in interactions) for the effective phenotyping of crop varieties for their responses to stem borers prior to deployment in farmers’ fields. Importantly, phenotyping results from experiments conducted under controlled conditions with two stem borer species were only weakly related to results from field plot experiments. In one case, a variety (T16) that appeared highly resistant in greenhouse and screen house experiments was heavily damaged in field plots likely because of interspecific interactions between two or three stem borer species. The two stem borer species showed marked differences in oviposition preferences and in their capacity to develop on rice at different growth stages. Varieties also differed in their capacities to compensate for damage from each of the stem borer species. These results highlight the need to devise robust phenotyping techniques and to determine optimal breeding systems – including a pre-emptive choice of desired traits – based on a firm knowledge of regional stem borer complexes, of interspecific stem borer interactions, and of the diversity of stemborer interactions with rice plants.

To date, over 50% of studies on rice-stem borer interactions have used whole plants during phenotyping experiments (based on 207 studies of varietal resistance; Horgan, unpublished). Such studies, that include natural and artificial infestations of rice plots, cannot differentiate between the responses by stem borers (sometimes more than one species) to different rice plant stages. Unless controls are included, they also confound rice resistance and tolerance. Different stem borer species have preferences for rice plants at specific growth stages. For example, the stalk-eyed fly, *D. longicornis,* occurs almost exclusively on seedlings (Alghali, 1983) and *S. innonata* on young, tillering plants (Rubia Sanchez et al., 1997). By infesting rice at tillering and booting stages, our greenhouse experiment demonstrated that both SSB and YSB performed better (greater survival, heavier pupae, etc.) on rice plants at vegetative stages compared to reproductive stages. In the same experiment, attacks by SSB at rice vegetative stages resulted in greater losses to yields than corresponding attacks at reproductive stages. The opposite occurred with YSB. However, the experiment may have overestimated the effects of stem borer damage on yields because we grew our rice plants in pots. Rice grown in pots has reduced tillering (Crisol et al., 2013); tillering is often a response and major compensation mechanism of rice plants to stem borer attacks at early vegetative stages (Rubia et al., 1996). During stem borer attacks, young, tillering rice plants can produce new tillers, or redirect nutrients to undamaged tillers. During late-stage attacks, plants can increase the proportion of productive tillers or increase grain weight (Rubia et al., 1996; Lv et al., 2008; Horgan et al., 2016). In our greenhouse experiment, compensation for stem borer damage was apparent among plants infested at the booting stage. Furthermore, the varieties IR64, IR66 and T16 overcompensated for late-stage damage from each moth species through increased grain yields. However, the mechanisms of overcompensation differed according to the attacking insect: when attacked by SSB, the varieties increased the proportion of grain that was filled, but when attacked by YSB, they increased grain weight.

In our wet- and dry-season field experiments, IR72 and IR66 had consistently lower damage. This may have been partly due to a low preference for these varieties by YSB during late tillering stages as indicated by low captures of adult YSB on these varieties during the dry season. However, in the screenhouse experiment, IR66 was the preferred host for YSB. Despite lower levels of damage, these two varieties sometimes had lower yields than the ‘high-damage’ varieties, IR68 and IR70, particularly during the dry season field trial. In the greenhouse, IR68 and IR70 compensated for damage, particularly when infested during the booting stage (i.e., based on biomass and grain for IR68; panicle number for IR70), which may have contributed to their maintenance of high yields in the face of heavy stem borer attacks. However, both IR68 and IR70 are also long-duration varieties (>125 days to mature) that had greater time to build up biomass and yield and to recover from damage in the greenhouse experiments. Both plants, but particularly IR68, are also relatively large (greater biomass), which is a trait often associated with tolerance (the ability to compensate for damage: Horgan et al., 2016; Horgan, 2017). Under field conditions, long-duration varieties incur more damage simply because they are exposed to stem borers for longer (*D. longicornis*: Alghali, 1983; *Chilo Agamemnon*: El-Malky et al., 2008; YSB: Bandong and Litsinger., 2005). As such, part of the interaction between IR68 or IR70 and rice stem borers might be better categorized as a ‘high vulnerability’ to damage (see Table 1). For example, in our screen house oviposition experiments using vegetative plants, neither of these plants (IR68 or IR70) appeared more susceptible than IR66 or IR72 because the experiments were terminated for all varieties at the same time. Furthermore, in the greenhouse experiment, all four varieties had similar reactions to SSB because this species had relatively poor development on reproductive tillers compared to YSB. Therefore, the incidence of whiteheads in field trials is an indicator of the combined effects of susceptibility, vulnerability and tolerance that requires further detailed tests to determine the contributions of each. In the same manner, whitehead damage is often not a good indicator of the relative impact of stem borers on yields (see also Rubia et al., 1989; Rubia Sanchez et al., 1997). Despite these observations, a low incidence of whiteheads is nevertheless a good parameter on which to base germplasm selection in breeding programs because whiteheads directly induce insecticide spraying by farmers (Bandong and Litsinger, 2005; Horgan, 2017) irrespective of normal levels of varietal tolerance to herbivory, which ultimately determines yields and yield losses. For rice breeders, varieties with low susceptibility/vulnerability combined with a high level of tolerance should be most desirable for regions prone to stem borer damage. Where damage from the stem borer complex is confined to early vegetative stages only (including in regions dominated by SSB), then long duration may be desired to allow increased plant recovery and compensation. Furthermore, depending on which rice stages are most vulnerable to attack by the dominant stem borers in a region, tolerance could be targeted for either tillering or reproductive stages.

Our experiments indicated field screening as the best method to select rice varieties with a low incidence of whiteheads – although this may result from several different plant traits (resistance, low vulnerability, or tolerance). Over 50% of studies designed to select stem borer ‘resistant’ rice varieties have conducted field screening. Most of these studies only report the incidence of dead heart or whitehead (based on 207 studies, Horgan, unpublished). A small number of previous studies have included yield losses from stem borers by comparing insecticide-treated and non-treated plots during screening (Catling et al., 1987; Kalode et al., 1987). Because stem borers occur as multispecies assemblages, interactions between species in the field could alter the outcome of single-species preferences and performance. This is determined not only by competition-based changes in the distribution of larvae during screening trials, but may also include interactions between the rice host and the natural enemies of stem borers (Rubia-Sanchez et al., 1997; Litsinger et al., 2006). Field screening can also uncover aspects of crop vulnerability that relate to climate, which is particularly beneficial as rice producing countries experience increasingly warmer temperatures under global climate change (Horgan, 2020). For example, temperate rice planted early in the season can escape stem borer damage (Bernhardt, 2003) – but asynchrony of planting in a landscape is known to increase damage – particularly in late-planted fields (Litsinger et al., 2006). Perhaps the greatest indicator of the value of field plots from our study was our successful selection of two varieties prone to high damage and two prone to relatively lower damage for our dry season study based on the results of the previous field season. Despite different seasons, different management practices and different field designs, results from both field experiments corresponded well. Meanwhile, the relative ranking of these four varieties was not apparent from restrictive experiments under controlled conditions.

A number of studies have noted the importance of tolerance in determining levels of damage and yield losses from stemborers in the field (Rubia et al., 1996; Rubia Sanchez et al., 1997; Horgan et al., 2016). However, it is difficult to phenotype for tolerance (Wise and Carr, 2008), which requires careful quantification of damage and the exclusion of intraspecific competition between the test herbivores (Horgan et al., 2018). To identify tolerance, researchers must also apply at least two levels of insect pressure (this may include zero damage) (Wise and Carr., 2008), and should observe ontogenetic changes in relative tolerance as the plants develop by including at least two stages in their study (Rubia et al., 1996; Bandong and Litsinger, 2005). In our experiments, we approximated tolerance by examining relative changes in plant condition (based on comparisons between infested and non-infested plants) under similar insect pressures; however, because stem borers reacted differently to the varieties, then the herbivore pressures on the plants varied across varietal treatments. Our estimates are therefore better regarded as relative condition changes that approximate tolerance, but more precise estimates of relative tolerance are difficult to attain. For field screening and other phenotyping tests, approximations of tolerance may be sufficient to indicate desirable rice genotypes (Rubia et al., 1996; Rubia Sanchez et al., 1997). Since tolerance and susceptibility are both affected by resource availability, then including at least two levels of a limiting resource, such as nitrogen, will also support phenotyping (Horgan et al., 2016, 2018). In our dry-season field study, the susceptibilities of IR68 and IR70 were most apparent at high nitrogen levels and there was a significant [variety × nitrogen] interaction because whitehead densities were the same for all four varieties at low nitrogen levels. In the same experiment, IR66 and IR72 showed marked responses to increasing nitrogen from 60 to 150 kg N ha^−1^, whereas IR68 in particular, did not. By applying nitrogenous fertilizer at ≥ 60 kg N ha^−1^ in both the wet and dry-season experiments, we could successfully rank varieties from low to high susceptibility, but this was not possible using lower fertilizer levels.

### 4.1 Recommendations

Based on our results we make the following recommendations during the phenotyping of rice varieties for their interactions with stem borers. Categories of rice-stem borer interaction should encompass relative measures from susceptibility to resistance, from high to low vulnerability, and from high to low tolerance. Escape from stem borers due to early planting (e.g., *Chilo plejadellus*: Bernhardt, 2003) cannot be regarded as a component of direct plant-based vulnerability, but crop duration is. Field trials, particularly if carried out in a hotspot, will expose test materials to realistic conditions including complete stem borer assemblages and interspecific stem borer interactions. Efficient screening of varieties for regional deployment is best achieved through field trials under high nitrogen conditions because stem borers will respond to high nitrogen through increased oviposition and fitness, but relative interaction categories are largely maintained across varieties. In effect, high fertilizer levels create local hotspots, particularly when poorly synchronized with surrounding rice fields. Finally, although high resistance and low vulnerability reduced stem borer damage by as much as 50% in our experiments, between 2% and 35% of tillers/plant were damaged even in varieties with a relatively low propensity for damage. Therefore, the selection of rice varieties based on comparatively low damage must be combined with other integrated pest management strategies, such as pheromone trapping, biological control, or farm diversification (Horgan, 2017; Horgan et al., 2019; Babendreier et al., 2020) to manage stem borers in farmers’ fields.

## Declaration of competing interest

The authors declare that they have no known competing financial interests or personal relationships that could have appeared to influence the work reported in this paper.
