# Supplementary material for: Stem borers revisited: Host resistance, tolerance, and vulnerability determine levels of field damage from a complex of Asian rice stemborers
Source: Crop Prot. 2021 Apr;142:105513. doi: 10.1016/j.cropro.2020.105513 (PMC7846815; doi:10.1016/j.cropro.2020.105513)
Supplement: Multimedia component 1 [file mmc1.docx]

SUPPLEMENTARY INFORMATION


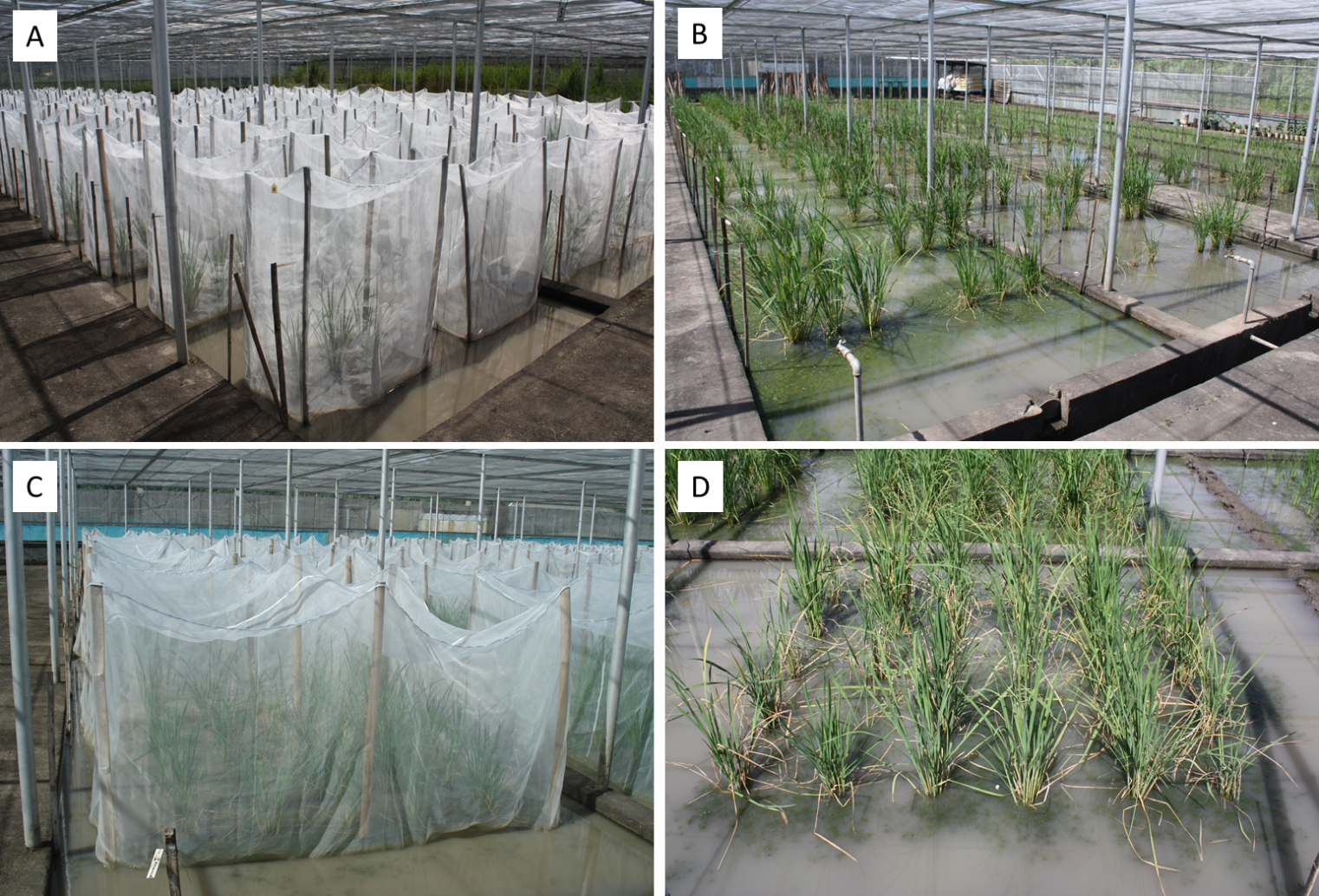


**Figure S1:** Experimental set-up for no-choice (A,B) and choice (C,D) experiments in the screenhouse facility. Note that the plants were transplanted to flooded-paddy soil in concrete bays. A and C indicate the cages with 3 plants (1 variety) and 30 plants (10 varieties), respectively. Cages have been removed in figures B and D.


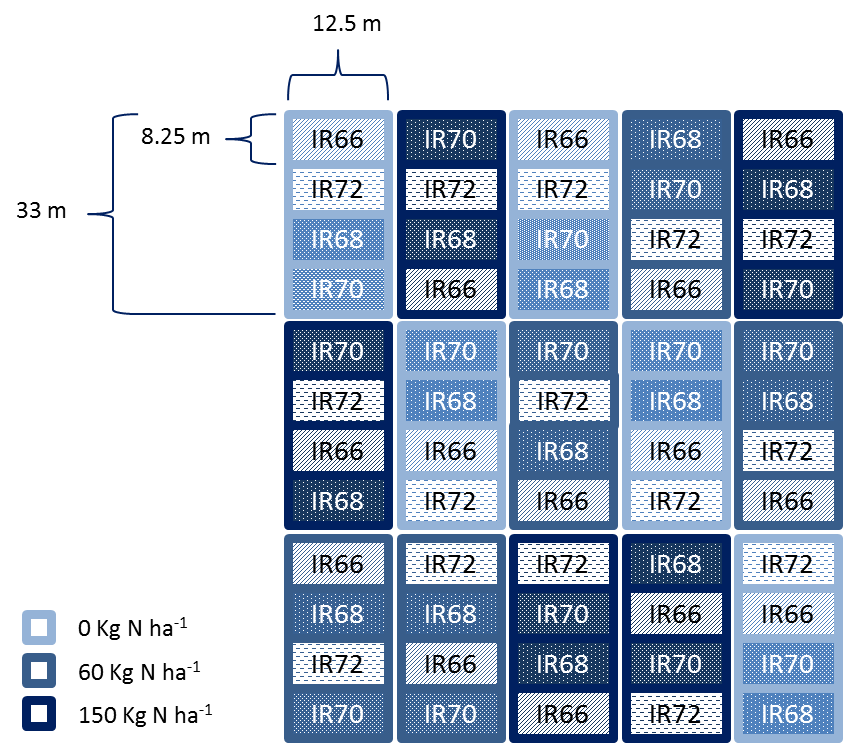


**Figure S2**: Field lay out for nitrogen experiment. Plots were treated with three levels of nitrogen and sub-plots planted with two susceptible (IR68, IR70) and two resistant (IR66, IR72) rice varieties.

**Table S1:** Result from dry-season screen house oviposition choice experiment with SSB

| Variety | Number of egg masses per plant^1^ | Proportion of egg masses on each plant^1^ | Number of larvae emerged on each plant^1^ | Proportion of larvae on each plant^1^ |
| --- | --- | --- | --- | --- |
| IR36 | 0.89 (0.27) | 0.10 (0.03) | 35.83 (13.82) | 0.11 (0.06) |
| IR40 | 1.17 (0.25) | 0.16 (0.03) | 38.67 (11.59) | 0.15 (0.06) |
| IR50 | 1.17 (0.19) | 0.20 (0.07) | 61.11 (20.54) | 0.22 (0.07) |
| IR62 | 1.00 (0.44) | 0.10 (0.05) | 57.61 (29.05) | 0.09 (0.05) |
| IR66 | 0.83 (0.28) | 0.09 (0.03) | 47.17 (23.38) | 0.10 (0.04) |
| IR68 | 0.78 (0.52) | 0.06 (0.03) | 44.61 (36.05) | 0.04 (0.03) |
| IR70 | 0.94 (0.42) | 0.08 (0.02) | 44.44 (33.79) | 0.04 (0.02) |
| IR72 | 0.83 (0.45) | 0.06 (0.02) | 52.33 (33.06) | 0.06 (0.03) |
| Taitung 16 | 0.50 (0.19) | 0.05 (0.01) | 36.44 (21.01) | 0.07 (0.03) |
| TKM6 | 1.22 (0.56) | 0.09 (0.03) | 60.38 (25.73) | 0.10 (0.03) |
| F-variety | 0.897ns | 0.897ns | 0.576ns | 0.576ns |

1: Results from GLM for F_9,50,_ data were ranked before analyses. ns = P > 0.05, numbers in parentheses are standard errors

**Table S2:** Changes in plant condition [mean (SEM)] under stem borer infestation based on results from the greenhouse experiment

| Species | stage | Rice line | Δ tillers (number)^1^ | Δ biomass (g) ^1^ | Δ panicles (number) ^1^ | Δ Grain (number) ^1^ | Δ filled grain (%)^1^ | Δ filled grain (g)^1^ | Δ filled grain (as proportion of control) ^2^ |
| --- | --- | --- | --- | --- | --- | --- | --- | --- | --- |
| SSB |  |  |  |  |  |  |  |  |  |
|  | Vegetative | IR36 | -0.67 (0.61)ab | -1.61 (1.49) | -1.67 (0.49)a | -13.82 (9.48)abc | -14.53 (11.14)ab | -0.47 (0.12)ab | -0.36 (0.10)ab |
|  |  | IR40 | 0.33 (0.67)ab | -5.90 (1.47) | -0.5 (0.67)ab | -33.05 (10.61)ab | -13.60 (7.20)ab | -0.57 (0.14)a | -0.49 (0.11)ab |
|  |  | IR50 | 1.17 (1.05)b | -2.01 (1.57) | 0.67 (1.31)ab | -35.81 (7.17)a | -2.21 (6.94)ab | -0.41 (0.16)ab | -0.28 (0.12)ab |
|  |  | IR62 | 0.33 (1.20)ab | -1.88 (1.17) | -0.5 (0.93)ab | -34.87 (11.96)abc | -5.40 (6.68)ab | -0.53 (0.12)ab | -0.33 (0.16)ab |
|  |  | IR64 | 1.00 (0.82)b | -2.30 (1.51) | -1.17 (0.86)ab | -20.86 (5.52)bc | 3.19 (13.02)b | -0.13 (0.07)bc | -0.15 (0.41)b |
|  |  | IR66 | 0.00 (1.48)b | -1.56 (1.55) | -2.00 (0.80)ab | -11.40 (16.17)abc | -6.74 (10.14)ab | -0.31 (0.20)abc | -0.13 (0.30)b |
|  |  | IR68 | 0.50 (1.48)b | -4.39 (1.95) | -1.00 (0.73)ab | -13.73 (8.14)abc | -6.59 (5.36)ab | -0.38 (0.25)ab | -0.04 (0.11)ab |
|  |  | IR70 | -1.67 (0.71)a | -6.08 (1.67) | 0.33 (0.58)b | -21.26 (15.06)abc | -8.84 (10.14)ab | -0.21 (0.23)abc | -0.10 (0.72)b |
|  |  | IR72 | -0.67 (0.56)ab | -3.00 (1.24) | -1.00 (0.65)ab | -3.10 (6.09)c | -18.71 (9.43)ab | -0.37 (0.12)abc | -0.35 (0.32)ab |
|  |  | *O. rufipogon* | -0.67 (0.80)b | -1.76 (3.09) | -0.83 (0.89)ab | -2.59 (5.97)abc | -19.70 (4.50)a | -0.27 (0.11)ab | -0.50 (0.09)a |
|  |  | Taitung 16 | 0.75 (0.16)b | -4.71 (1.57) | -1.00 (0.65)ab | 0.21 (5.19)c | 5.19 (3.33)b | 0.38 (0.20)c | 1.47 (0.11)ab |
|  |  | TKM6 | 0.00 (1.15)ab | -0.57 (3.35) | -1.17 (0.75)ab | -21.75 (6.62)abc | 6.13 (9.08)ab | -0.08 (0.22)ab | 0.28 (0.17)ab |
|  | Reproductive | IR36 | -4.50 (0.85) | -2.88 (1.35) | -1.67 (0.92) | 9.83 (9.03) | -17.01 (3.40) | -0.14 (0.14) | -0.10 (0.11) |
|  |  | IR40 | -4.80 (0.70) | -6.32 (0.66) | 0.00 (.930 | -15.54 (5.91) | -12.08 (1.70) | -0.36 (0.08) | -0.31 (0.06) |
|  |  | IR50 | -1.15 (1.02) | -2.19 (1.91) | 1.00 (0.86) | -23.71 (5.29) | 6.60 (5.66) | -0.10 (0.10) | -0.04 (0.11) |
|  |  | IR62 | -1.67 (0.80) | -1.93 (0.67) | 1.00 (0.550 | -2.56 (9.98) | 0.60 (6.960 | -0.10 (0.19) | 0.08 (0.19) |
|  |  | IR64 | -0.50 (1.28) | -2.20 (1.49) | 0.00 (0.40) | 21.72 (7.67) | 8.40 (9.56) | 0.32 (0.11) | 0.58 (0.51) |
|  |  | IR66 | -0.60 (0.33) | -1.82 (1.60) | -0.40 (0.70) | 1.02 (13.48) | 1.61 (7.35) | 0.04 (0.17) | 0.27 (0.14) |
|  |  | IR68 | -1.55 (1.06) | 2.63 (3.10) | 1.17 (1.05) | 7.73 (5.95) | -12.91 (5.51) | -0.29 (0.23) | 0.15 (0.21) |
|  |  | IR70 | -5.83 (0.70) | -2.92 (1.96) | 1.83 (0.80) | -2.05 (8.22) | 4.65 (10.75) | 0.03 (0.09) | 0.10 (0.70) |
|  |  | IR72 | 3.00 (1.32) | -0.23 (1.04) | 1.33 (0.73) | 14.22 (9.02) | -6.17 (6.10) | 0.04 (0.18) | 0.15 (0.19) |
|  |  | *O. rufipogon* | 1.00 (0.82) | 1.34 (2.88) | -0.17 (0.82) | -3.74 (7.36) | -24.58 (5.87) | -0.27 (0.07) | -0.61 (0.25) |
|  |  | Taitung 16 | 0.17 (0.65) | -1.30 (2.91) | 0.33 (0.26) | 13.73 (4.83) | 5.75 (4.22) | 0.23 (0.16) | 1.00 (0.23) |
|  |  | TKM6 | -2.33 (0.76) | -1.36 (3.21) | 0.00 (0.60) | -17.33 (4.81) | -7.77 (4.960 | -0.42 (0.18) | -0.39 (0.04) |
|  | F-rice line (V)^3^ |  | 3.952*** | 1.306ns | 2.009* | 3.605*** | 2.733** | 3.406*** | 3.381*** |
|  | F-plant stage (P) ^4^ |  | 31.131*** | 2.826ns | 14.031*** | 24.466*** | 0.608ns | 8.610*** | 13.067*** |
|  | V × P^3^ |  | 1.889* | 0.761ns | 0.417ns | 0.859ns | 0.592ns | 1.194ns | 1.814ns |
| YSB |  |  |  |  |  |  |  |  |  |
|  | Vegetative | IR36 | -1.33 (1.15)abc | -0.56 (1.34)ab | -1.67 (0.56)ab | -21.93 (8.54)ab | -10.37 (5.88) | -0.44 (0.11) | -0.34 (0.08)ab |
|  |  | IR40 | -1.83 (0.87)a | -4.75 (0.86)ab | -0.50 (0.72)a | -25.25 (6.84)ab | -13.51 (5.00) | -0.50 (0.11) | -0.44 (0.10)ab |
|  |  | IR50 | 1.40 (1.02)bc | -2.95 (1.30)ab | -0.40 (1.05)ab | -56.99 (6.09)a | 0.61 (5.64) | -0.57 (0.07) | -0.43 (0.06)ab |
|  |  | IR62 | -2.00 (1.84)abc | -1.80 (1.08)ab | -0.33 (0.83)ab | -39.50 (10.00)ab | -10.90 (8.42) | -0.63 (0.25) | -0.32 (0.26)ab |
|  |  | IR64 | 0.33 (1.43)bc | -2.48 (1.88)ab | -1.00 (0.79)ab | -19.89 (7.23)ab | 0.78 (6.32) | -0.11 (0.10) | -0.15 (0.19)ab |
|  |  | IR66 | 0.31 (0.81)bc | -0.94 (0.98)ab | -1.19 (0.20)ab | -14.86 (3.15)ab | -0.12 (8.05) | -0.23 (0.24) | 0.11 (0.13)b |
|  |  | IR68 | 1.00 (1.00)bc | -3.45 (1.75)b | -1.80 (0.66)ab | 1.92 (9.81)b | -0.66 (6.97) | -0.07 (0.18) | 0.04 (0.15)ab |
|  |  | IR70 | -1.42 (0.20)ab | -2.11 (1.42)ab | 0.42 (0.71)b | -13.03 (23.61)ab | -13.78 (9.90) | -0.31 (0.12) | -0.33 (0.37)ab |
|  |  | IR72 | -2.20 (0.54)abc | -2.75 (0.70)ab | -0.60 (0.75)ab | -12.50 (20.82)ab | 0.54 (9.67) | -0.07 (0.16) | -0.03 (0.29)ab |
|  |  | *O. rufipogon* | 0.33 (0.88)c | -3.34 (2.24)ab | -2.33 (0.61)a | -7.13 (12.63)ab | -8.89 (9.57) | -0.24 (0.13) | -0.36 (0.24)a |
|  |  | Taitung 16 | 0.00 (0.58)abc | -11.48 (2.77)a | -2.67 (1.39)ab | -30.96 (7.39)ab | -26.00 (5.14) | -0.19 (0.22) | -0.51 (0.14)ab |
|  |  | TKM6 | -1.00 (0.89)abc | -4.80 (2.36)ab | -1.83 (0.80)ab | -44.04 (11.24)a | 5.29 (14.14) | -0.24 (0.30) | -0.25 (0.21)ab |
|  | Reproductive | IR36 | -4.20 (0.40) | -2.90 (1.20) | -0.60 (0.20) | -4.26 (9.86) | -14.16 (4.01) | -0.33 (0.11) | -0.24 (0.08) |
|  |  | IR40 | -5.67 (0.61) | -5.14 (1.40) | -2.33 (0.99) | 3.17 (5.75) | -35.78 (5.13) | -0.47 (0.06) | -0.42 (0.05) |
|  |  | IR50 | -1.50 (1.15) | 0.12 (1.40) | 0.83 (1.01) | -9.37 (8.50) | -13.35 (4.22) | -0.31 (0.17) | -0.17 (0.17) |
|  |  | IR62 | -0.83 (1.19) | 0.68 (0.87) | 1.50 (0.55) | -7.08 (16.68) | -16.18 (9.99) | -0.41 (0.17) | -0.21 (0.48) |
|  |  | IR64 | -0.67 (1.12) | -0.19 (2.38) | 0.67 (0.76) | 23.48 (5.01) | -4.16 (8.32) | 0.11 (0.18) | 0.28 (0.38) |
|  |  | IR66 | -1.20 (0.79) | -0.84 (0.97) | -1.40 (0.95) | 13.65 (9.68) | -10.43 (8.83) | -0.05 (0.38) | 0.50 (0.15) |
|  |  | IR68 | -1.40 (1.23) | 3.61 (2.18) | 0.40 (0.66) | 9.92 (7.74) | -26.41 (9.11) | -0.61 (0.22) | -0.32 (0.22) |
|  |  | IR70 | -5.00 (0.93) | 0.14 (1.74) | 2.20 (0.80) | -5.52 (8.44) | -21.35 (9.03) | -0.44 (0.15) | -0.47 (0.41) |
|  |  | IR72 | -3.40 (1.25) | -1.16 (2.52) | 1.40 (0.70) | -9.20 (8.29) | -20.72 (5.23) | -0.44 (0.24) | -0.27 (0.19) |
|  |  | *O. rufipogon* | 0.50 (0.50) | 0.39 (1.52) | -0.83 (1.06) | -19.04 (6.31) | -23.64 (6.27) | -0.35 (0.10) | -0.73 (0.16) |
|  |  | Taitung 16 | -1.17 (0.60) | -1.44 (2.55) | 0.67 (0.33) | 22.50 (4.46) | -3.87 (9.02) | 0.18 (0.17) | 0.39 (0.31) |
|  |  | TKM6 | -2.17 (0.48) | -0.29 (3.81) | 1.17 (0.75) | -18.54 (8.72) | -12.57 (7.77) | -0.38 (0.15) | -0.42 (0.06) |
|  | F-rice line (V)^3^ |  | 4.348*** | 2.015* | 2.680** | 2.703** | 1.381ns | 1.690ns | 1.993* |
|  | F-plant stage (P)^4^ |  | 18.386*** | 14.262*** | 20.669*** | 30.053*** | 10.459*** | 0.014ns | 0.689ns |
|  | V × P^3^ |  | 1.191ns | 1.539ns | 1.520ns | 1.708ns | 1.263ns | 1.089ns | 1.286ns |

1: calculated as infested – control

2: calculated as (infested-control)/control

1,2: Numbers in parentheses are standard errors. Lowercase letters indicate homogenous variety groups (Tukey test, P < 0.05). Note that homogenous groups are calculated using the combined results for vegetative and reproductive plants and are indicated only once per variety/wild rice species for each stemborer species

3: DF = 11,120, *** = P ≤ 0.005; ** = P ≤ 0.01; * = P ≤ 0.05; ns = P > 0.05

4: DF = 1,120, *** = P ≤ 0.005; ** = P ≤ 0.01; * = P ≤ 0.05; ns = P > 0.054: Data ranked before analysis

**Table S3:** Traits of healthy – non-infested, rice plants from field plots with ten rice varieties

| Sample | Variety | Number of tillers/plant | Plant height (cm) | Leaves/tiller | Shoot biomass (g dry wgt) | Leaf biomass (g dry wgt)/tiller | Panicle biomass (g dry weight) | Weight of filled grain (g) | Weight of unfilled grain (g) | 100 filled grain weight | 100 unfilled grain weight |
| --- | --- | --- | --- | --- | --- | --- | --- | --- | --- | --- | --- |
| Early September |  |  |  |  |  |  |  |  |  |  |  |
|  | IR36 | 51.88 (1.73) | 81.73 (1.92)b | 4.24 (0.08)ab | 17.10 (1.06)bc | 11.86 (0.46)ab |  |  |  |  |  |
|  | IR40 | 51.13 (1.19) | 71.96 (2.51)c | 3.92 (0.04)b | 13.92 (0.80)cd | 10.13 (0.38)ab |  |  |  |  |  |
|  | IR50 | 48.08 (2.97) | 77.63 (1.34)a | 4.04 (0.03)b | 17.96 (1.09)a | 9.92 (0.82)ab |  |  |  |  |  |
|  | IR62 | 40.83 (1.63) | 84.76 (1.40)c | 4.41 (0.05)bc | 17.34 (1.06)b | 11.45 (0.53)ab |  |  |  |  |  |
|  | IR66 | 43.71 (3.77) | 82.88 (2.05)c | 4.29 (0.06)b | 18.16 (1.71)bcd | 13.85 (1.36)ab |  |  |  |  |  |
|  | IR68 | 32.75 (1.91) | 95.64 (1.94)d | 4.45 (0.05)c | 17.75 (0.91)cd | 14.32 (1.05)b |  |  |  |  |  |
|  | IR70 | 44.71 (1.76) | 78.79 (2.25)c | 4.13 (0.08)bc | 16.58 (0.96)d | 11.86 (1.20)ab |  |  |  |  |  |
|  | IR72 | 32.96 (1.23) | 80.89 (1.18)b | 4.60 (0.04)bc | 22.49 (1.40)bcd | 9.78 (0.22)ab |  |  |  |  |  |
|  | T16 | 20.04 (1.43) | 95.12 (1.65)d | 4.64 (0.06)b | 16.58 (0.71)bcd | 9.50 (0.41)ab |  |  |  |  |  |
|  | TKM6 | 46.21 (1.76) | 106.78 (3.17)e | 3.98 (0.03)a | 18.36 (0.96)bcd | 11.01 (0.19)a |  |  |  |  |  |
| Late September |  |  |  |  |  |  |  |  |  |  |  |
|  | IR36 | 29.54 (1.25) | 98.78 (1.02) | 4.12 90.07) | 56.02 (2.88) | 3.12 (0.00) | 5.54 (5.54) |  |  |  |  |
|  | IR40 | 35.71 (1.47) | 114.73 (1.55) | 4.74 (0.04) | 67.33 (3.43) | 10.00 | 0.00 (0.00) |  |  |  |  |
|  | IR50 | 40.38 (4.96) | 83.77 (0.58) | 3.88 (0.07) | 17.99 (1.35) | 9.08 (0.95) | 13.23 (1.28) |  |  |  |  |
|  | IR62 | 29.04 (1.43) | 112.78 (1.88) | 4.74 (0.06) | 40.47 (6.61) | 12.13 (0.22) | 19.01 (6.26) |  |  |  |  |
|  | IR66 | 28.54 (0.97) | 107.57 (1.58) | 4.53 (0.06) | 59.75 (7.92) | 12.81 (1.31) | 30.39 (9.95) |  |  |  |  |
|  | IR68 | 20.29 (1.10) | 118.92 (3.37) | 5.25 (0.07) | 64.47 (3.21) | 10.00 | 0.00 (0.00) |  |  |  |  |
|  | IR70 | 27.29 (1.02) | 114.08 (1.02) | 4.91 (0.08) | 70.34 (3.11) | 10.00 | 0.00 (0.00) |  |  |  |  |
|  | IR72 | 24.79 (1.06) | 95.71 (1.84) | 4.19 (0.09) | 59.50 (3.85) | 8.68 (0.48) | 12.05 (7.64) |  |  |  |  |
|  | T16 | 18.79 (0.48) | 119.25 (0.61) | 4.25 (0.13) | 57.48 (3.66) | 5.99 (0.00) | 1.87 (1.87) |  |  |  |  |
|  | TKM6 | 36.42 (2.79) | 129.03 (1.35) | 3.91 (0.06) | 55.22 (2.39) | 10.00 | 4.19 (4.19) |  |  |  |  |
| Harvest |  |  |  |  |  |  |  |  |  |  |  |
|  | IR36 | 30.58 (1.18) | 97.03 (1.34) | 4.01 (0.17) | 17.32 (0.75) | 10.75 (0.54) | 28.82 (1.41)bc | 1217.29 (41.58)d | 142.42 (32.92)ab | 1.95 (0.06)b | 0.52 (0.11) |
|  | IR40 | 27.29 (1.26) | 113.27 (1.30) | 3.99 (0.18) | 30.48 (1.45) | 15.08 (0.63) | 15.16 (1.59)a | 953.92 (43.53)c | 124.92 (14.24)ab | 1.92 (0.06)b | 0.41 (0.02) |
|  | IR50 | 54.56 (9.28) | - | - | 18.85 (1.29) | 9.83 (1.12) | 14.63 (1.38)a | 347.03 (65.39)a | 132.34 (15.44)ab | 1.43 (0.02)a | 0.37 (0.06) |
|  | IR62 | 27.63 (1.28) | 114.15 (2.18) | 3.86 (0.18) | 27.50 (1.40) | 15.97 (0.89) | 21.45 (1.73)ab | 1147.79 (58.53)cd | 173.17 (14.79)b | 2.05 (0.05)b | 0.36 (0.04) |
|  | IR66 | 27.46 (1.40) | 106.54 (1.57) | 3.76 (0.13) | 22.08 (1.24) | 14.70 (0.84) | 28.89 (2.52)bc | 1308.92 (17.62)d | 114.20 (7.86)ab | 1.91 (0.02)b | 0.43 (0.05) |
|  | IR68 | 17.42 (1.05) | 118.98 (1.85) | 4.22 (0.20) | 31.78 (1.61) | 18.98 (1.22) | 16.72 (1.14)a | 920.06 (34.46)bc | 160.97 (18.36)b | 2.73 (0.14)d | 0.58 (0.04) |
|  | IR70 | 22.58 (0.89) | 113.63 (1.84) | 4.11 (0.18) | 31.02 (1.31) | 15.89 (1.07) | 16.09 (1.15)a | 1240.46 (25.82)d | 151.02 (16.62)ab | 1.95 (0.02)b | 0.38 (0.05) |
|  | IR72 | 24.96 (1.75) | 93.68 (1.98) | 4.32 (0.27) | 21.01 (1.18) | 11.11 (1.05) | 34.65 (2.27)c | 1383.28 (52.77)d | 76.34 (12.43)a | 2.15 (0.02)bc | 0.38 (0.04) |
|  | T16 | 18.08 (0.91) | 117.93 (1.59) | 3.86 (0.12) | 23.59 (1.01) | 12.15 (0.59) | 22.36 (1.40)ab | 922.85 (44.75)bc | 120.70 (9.67)ab | 2.38 (0.08)c | 0.53 (0.10) |
|  | TKM6 | 38.13 (4.70) | 123.23 (2.53) | 3.61 (0.15) | 28.59 (2.66) | 10.66 (0.96) | 14.33 (2.71)a | 695.10 (102.26)b | 150.70 (18.66)ab | 1.56 (0.02)a | 0.57 (0.17) |
| F-sampling date (T)^2^ |  | 160.208*** | 714.358*** | 29.193*** | 604.328*** | 22.778*** |  |  |  |  |  |
| F-T × variety^2^ |  | 6.417*** | 14.382*** | 7.909*** | 12.245*** | 2.187ns |  |  |  |  |  |
| F-variety^3^ |  | 31.387*** | 74.253*** | 9.178*** | 12.534*** | 4.071** | 15.975*** | 34.837*** | 601.389*** | 37.559*** | 1.194ns |

1: Standard errors indicated in parentheses; lowercase letters indicate homogenous variety groups.

2: Within factor sampling date DF = 2,100; T × variety DF = 18,100; ns= P > 0.05, *** = P ≤ 0.001; Tiller number log+1 transformed

3: Between factor variety DF = 9,50; ns= P > 0.05, ** = P ≤ 0.01, *** = P ≤ 0.001; Tiller number log+1 transformed

**Figure S3**: Biplots of rice traits (based on non-infested plants) and stem borer damage (black symbols) during the vegetative (A-F) and reproductive (G-M) stages of rice crop development. Traits were (A,G) tiller number, (B,H) tiller weight, (C,I) shoot weight, (D,J) leaf weight, (E,K) plant height, (F,L) leaves per tiller, and (M) panicle weight.

**Table S4:** Results from dry season field experiment on the effects of nitrogen and variety on plant development and stem borer incidence in field plots. Numbers are means (SEM) based on harvest samples

| Variety | Nitrogen level (Kg ha^-1^) | Tiller number | Plant height (cm) | Plant biomass (g dry weight) | Root biomass (g dry weight) | Shoot biomass (g dry weight) | Panicle weight (g dry weight) | Filled grain  (g dry weight) | Unfilled grain (g dry weight) | Whiteheads per plant | Adult moths (at 60 DAT) per sweepnet sample |
| --- | --- | --- | --- | --- | --- | --- | --- | --- | --- | --- | --- |
| IR66 | 0 | 20.20 (2.48)aAB | 81.64 (2.62)A | 57.94 (9.00)aA | 3.15 (0.77)A | 26.75 (4.69)aA | 1.66 (0.26)aAB | 21.93 (3.22)aA | 1.86 (0.47)abA | 0.00 (0.00)aA | 0.00 (0.00)aA |
| IR66 | 60 | 23.00 (1.64)a | 82.04 (1.62) | 69.31 (4.37)a | 3.30 (0.24) | 31.81 (1.21)a | 3.88 (0.39)ab | 26.28 (2.60)b | 1.44 (0.21)a | 0.00 (0.00)a | 0.00 (0.00)a |
| IR66 | 150 | 26.60 (1.12)b | 85.38 (1.51) | 87.52 (6.97)b | 3.89 (0.34) | 38.79 (1.77)b | 4.69 (0.80)b | 34.81 (4.91)b | 2.62 (1.02)b | 0.20 (0.20)b | 0.00 (0.00)b |
| IR68 | 0 | 20.80 (2.08)A | 95.24 (1.66)C | 111.25 (16.20)C | 5.79 (0.71)C | 53.89 (7.93)B | 2.77 (0.43)B | 42.79 (7.04)B | 3.03 (0.93)B | 0.60 (0.24)B | 0.20 (0.20)B |
| IR68 | 60 | 18.60 (1.12) | 101.82 (2.53) | 112.45 (3.64) | 5.25 (0.73) | 51.49 (3.38) | 3.08 (0.07) | 47.24 (0.73) | 1.96 (0.32) | 0.00 (0.00) | 0.00 (0.00) |
| IR68 | 150 | 21.40 (1.57) | 110.28 (2.26) | 125.32 (5.63) | 5.41 (0.55) | 58.88 (3.65) | 3.56 (0.21) | 50.16 (4.20) | 3.47 (0.42) | 1.00 (0.32) | 0.00 (0.00) |
| IR70 | 0 | 23.80 (1.53)AB | 84.68 (3.73)A | 89.26 (7.07)B | 4.51 (0.50)BC | 48.03 (3.49)B | 2.52 (0.20)B | 29.15 (3.07)A | 2.64 (0.27)AB | 0.00 (0.00)B | 0.80 (0.80)B |
| IR70 | 60 | 23.80 (0.86) | 89.78 (2.25) | 92.42 (4.15) | 4.32 (0.54) | 46.15 (1.72) | 2.86 (0.23) | 34.40 (2.10) | 2.09 (0.31) | 0.60 (0.24) | 0.00 (0.00) |
| IR70 | 150 | 24.20 (2.15) | 94.60 (2.77) | 102.39 (7.40) | 5.27 (0.54) | 53.65 (4.38) | 3.33 (0.38) | 34.85 (2.64) | 2.76 (0.41) | 0.80 (0.37) | 0.00 (0.00) |
| IR72 | 0 | 17.60 (0.68)B | 74.16 (1.70)B | 53.96 (4.77)A | 3.07 (0.37)AB | 26.27 (2.53)A | 2.80 (0.88)A | 17.81 (1.93)A | 1.32 (0.13)AB | 0.00 (0.00)A | 0.00 (0.00)A |
| IR72 | 60 | 23.60 (2.06) | 77.68 (2.01) | 75.27 (8.44) | 4.10 (0.70) | 37.16 (4.15) | 1.70 (0.49) | 28.56 (4.15) | 1.80 (0.25) | 0.00 (0.00) | 0.00 (0.00) |
| IR72 | 150 | 33.20 (3.26) | 88.44 (2.64) | 97.73 (8.31) | 4.40 (0.48) | 47.63 (6.38) | 3.34 (0.86) | 36.46 (1.55) | 3.41 (0.72) | 0.00 (0.00) | 0.00 (0.00) |
| Field/block^1^ | 4 | 1.656ns | 0.385ns | 2.363ns | 5.990* | 2.634ns | 4.773* | 1.731ns | 8.059*** | 18.765*** | 9.566** |
| Nitrogen (N)^2^ | 2 | 10.664** | 2.521ns | 12.651** | 3.443ns | 10.744** | 26.885*** | 16.943*** | 38.400*** | 83.601*** | 8.206* |
| Variety (V)^3^ | 3 | 31.850*** | 56.819*** | 221.865*** | 178.628*** | 241.1747*** | 47.890*** | 298.056*** | 87.893*** | 1023.898*** | 51.141*** |
| N × V^4^ | 6 | 35.928*** | 1.445ns | 0.957ns | 13.219*** | 12.4309*** | 81.932*** | 12.015*** | 3.597*** | 425.0112*** | 13.010*** |

1: Field/block DF = 4,8; ns= P > 0.05, * = P ≤ 0.05, ** = P ≤ 0.01, *** = P ≤ 0.001

2: Nitrogen DF = 2,8; ns= P > 0.05, * = P ≤ 0.05, ** = P ≤ 0.01, *** = P ≤ 0.001; Uppercase letters indicate homogenous nitrogen groups.

3: Variety (subplot) DF = 3,24; *** = P ≤ 0.001; lowercase letters indicate homogenous variety groups.

4: N × V DF = 6,24; ns= P > 0.05, *** = P ≤ 0.001
